# Drug-induced gastrointestinal toxicity and barrier integrity: cytoskeleton-mediated impairment in a clinically relevant human intestinal epithelium model

**DOI:** 10.1038/s12276-025-01635-6

**Published:** 2026-02-12

**Authors:** Won Dong Yu, Sugi Lee, Hyun-Soo Cho, Ohman Kwon, Jung Hwa Lim, Cho-Rok Jung, Byunghyun Jang, Kyung Jin Lee, Jongman Yoo, Dae-Soo Kim, Hana Lee, Mi-Young Son

**Affiliations:** 1 Korea Research Institute of Bioscience and Biotechnology, Daejeon, Korea; 2 KRIBB School of Bioscience, Korea University of Science and Technology, Daejeon, Korea; 3 ORGANOIDSCIENCES, Seongnam, Republic of Korea; 4 Lambda Biologics, Leipzig, Germany; 5 CHA Organoid Research Center, CHA University, Seongnam, Republic of Korea; 6 School of Medicine, Sungkyunkwan University, Suwon, Republic of Korea

**Keywords:** Embryonic stem cells, High-throughput screening, Stress fibres

## Abstract

Drug-induced gastrointestinal (GI) toxicity is common, dose-limiting and difficult to predict using conventional Caco-2-based assays that lack physiological relevance. Here we evaluate a transepithelial electrical resistance (TEER) assay using nontransformed human intestinal epithelial cells (hIECs), derived from human pluripotent stem cells, which superiorly recapitulated epithelial diversity and polarity as well as intestinal barrier function. Across 17 clinically relevant compounds (cell cycle inhibitors, tyrosine kinase inhibitors and nonsteroidal anti-inflammatory drugs), the hIEC TEER assay outperformed ATP cell viability assays, and the Caco-2 TEER assay (AUC of 0.96 for hIEC TEER, 0.72 for Caco-2 TEER and ≤0.69 for cell viability assays) correlated with integrated GI toxicity scores using a ≥50% TEER reduction cutoff (sensitivity 92%, specificity 100% and accuracy 94%). Drug exposure was quantified by calculating the margin of safety (IC_15_:*C*_max_) and a lumen–surrogate margin of safety for oral agents. For mechanistic insight, transcriptomic analysis using representative chemotherapeutics (paclitaxel and docetaxel) showed the downregulation of cytoskeleton-related pathways, including cytoskeleton in muscle cells, cell adhesion molecules and extracellular matrix–receptor interaction, linking microtubule-targeting chemotherapy to intestinal barrier impairment. This platform provides a robust tool that combines predictive accuracy with the evaluation of cytoskeleton-mediated barrier impairment, enabling the early identification of drug-induced GI toxicity.

## Human intestinal cells enhance drug toxicity predictions

Clinical trials often fail owing to drug toxicity causing symptoms such as diarrhea and abdominal pain. This study addresses the need for better methods to predict gastrointestinal (GI) toxicity. Researchers developed a new test using human intestinal epithelial cells derived from stem cells. They compared this with the widely used Caco-2 model. The researchers used a method called transepithelial electrical resistance to measure how well the intestinal barrier functioned after drug exposure. This method is important because it can detect early signs of barrier disruption, a key indicator of GI toxicity, often before standard viability assays show damage. The results showed that the human intestinal epithelial cell-based transepithelial electrical resistance assay was more accurate in predicting drug-induced GI toxicity than traditional methods. The study concludes that this new model could strengthen preclinical safety testing and improve drug development by reducing clinical trial failures. This summary was initially drafted using artificial intelligence, then revised and fact-checked by the author.

## Introduction

Clinical trials fail primarily because of inaccurate predictions of drug toxicity, a lack of clinical efficacy, adverse drug effects and financial constraints^1,2^. Intestinal toxicity is one of the most overt manifestations of drug administration and substantially reduces the quality of life. The primary symptoms include vomiting, abdominal pain, diarrhea, nausea, inflammation, ulcers, immune suppression and nutrient deficiencies^3–6^. Gastrointestinal (GI) toxicity ranks fourth among clinical failures, following neurotoxicity, cardiovascular toxicity and hepatotoxicity^7^. These data highlight the urgent need for specific and sensitive evaluation methods based on models that can predict GI toxicity to improve the safety profiles of existing and investigational new drugs and help prevent drug-induced toxicity.

Historically, drug cytotoxicity has been assessed using cell viability assays that measure ATP and NADPH concentrations^8–10^. These assays can evaluate cellular responses but cannot accurately distinguish between true cytotoxicity and metabolic fluctuations induced by drug treatment. In addition, because the intestinal epithelium has robust tight junction complexes, barrier dysfunction often precedes cell death^3,5,11^, underscoring the need for methods that can detect barrier integrity disruption rather than relying solely on cell viability assays.

The Caco-2 cell line is widely used as a model of human intestinal epithelial cells (hIECs) primarily owing to its ability to differentiate into absorptive enterocyte-like cells with microvilli, tight junctions and enzymatic activity^12–14^. However, Caco-2 cells are immortalized human colorectal adenocarcinoma cells that lack specialized intestinal cell types and metabolic functions, exhibiting transepithelial electrical resistance (TEER) values that differ markedly from those of native intestinal tissue. Thus, Caco-2 cells and conventional three-dimensional (3D) organoids exhibit limited physiological relevance for oral absorption and toxicity studies owing to reduced intestinal cell diversity, limited metabolic capacity, atypical drug-metabolizing and transporter profiles and nonphysiological barrier functions. Moreover, organoids restrict access to the apical surface, preventing realistic dosing. An ideal in vitro intestinal model for assessing drug-induced toxicity should accurately reflect critical GI parameters (epithelial membrane tightness, mucosal structure, epithelial transporter expression and metabolic enzyme profiles) because these factors substantially influence drug absorption and tissue responses^15^. Evaluating barrier integrity using these parameters can help predict GI toxicity.

We previously derived normal hIECs from human pluripotent stem cells (hPS cells); hIECs demonstrated greater physiological relevance than traditional Caco-2 cell models^16^. In addition, this hIEC model captured key GI parameters, including barrier integrity, apical–basolateral polarity, the presence of mucus-secreting cells, diverse epithelial transporter expression and enhanced metabolic enzyme activities.

Cytoskeletal organization is central to maintaining intestinal barrier function as it underpins tight junction stability, cell adhesion and mechanical integrity^17^. The disruption of cytoskeletal systems underlies barrier disruption and increased intestinal permeability in various pathological conditions^18,19^. Microtubule-targeting agents such as paclitaxel and docetaxel interfere with cytoskeletal integrity^20,21^; nonetheless, their impact on the transcriptional regulation of cytoskeleton-related genes in normal human intestinal epithelium remains poorly characterized. Integrating functional TEER measurements with transcriptomic profiling enables us to correlate drug-induced barrier changes with underlying cytoskeletal alterations.

In this study, we validated the predictive accuracy of the hIEC-based TEER assay by evaluating 17 clinically relevant drugs from three pharmacological classes associated with GI toxicity. Our model provides a robust platform for predicting drug-induced intestinal toxicity, combining high predictive accuracy with the mechanistic evaluation of cytoskeleton-mediated barrier impairment, thereby guiding preclinical development.

## Materials and methods

### Generating hIEC progenitors from hPS cells

This study was approved by the Korean Public Institutional Review Board (approval numbers P01-201409-ES-01 and P01-201609-31-002). hPS cells, including the H9 hESC line (WA09, WiCell Research Institute) (before passage 60) and induced pluripotent stem cells, were cultured as described previously^22,23^. Expandable hIEC progenitors were generated from hPS cells as previously described.

The hIEC progenitors were cultured in hIEC medium 1 containing DMEM/F12 (Thermo Fisher Scientific, no. 11330), 2% fetal bovine serum (FBS), 1% penicillin–streptomycin (Gibco, no. 15140-122), 2 mM L-glutamine (Gibco, no. 25030-081), 15 mM HEPES (Gibco, no. 15630-080), 2% B27 supplement (Thermo Fisher Scientific, no. 12587-001), 1% N2 supplement (Thermo Fisher Scientific), epidermal growth factor (EGF) (100 ng/ml; R&D Systems, no. 236-EG-01M), R-spondin 1 (200 ng/ml; Peprotech, no. 120-8) and insulin (5 μg/ml; Sigma, no. I9278). hIEC medium 1 was replaced every other day. Seven days after seeding, hIEC progenitors were dissociated using trypsin–EDTA (Thermo Fisher Scientific, no. 25300062) for 5 min at 37 °C. The cell pellet was resuspended in fresh hIEC medium 1 and reseeded onto 1% Matrigel-coated plates at a density of 1.34 × 10^6^ cells/cm^2^. On the first day, 10 μM Y-27632 (R&D Systems, no. 1254/10) was used to enhance cell survival. For cryopreservation, hIEC progenitors were gently dissociated, slowly frozen in CryoStor CS10 freezing medium (Stemcell, no. ST100-1061) and stored in liquid nitrogen at −196 °C.

### Differentiation of hIEC progenitors into functional hIECs

To establish a mature and functional intestinal epithelium model, hIEC progenitors were seeded onto 1% Matrigel-coated Transwell inserts (Corning, no. 3460) at a density of 1.34 × 10^5^ cells/cm^2^ in hIEC medium 1 containing 10 μM Y-27632. After confluence, the medium was replaced with differentiation medium (hIEC medium 2) containing DMEM/F12 supplemented with EGF (100 ng/ml), 2 μM Wnt-C59 (Selleckchem, no. S7037), 1 mM valproic acid (Sigma, no. P4543), 2% FBS, 2% B27 supplement, 1% N2 supplement, 2 mM L-glutamine, 1% nonessential amino acids (Thermo Fisher Scientific, no. 11140050) and 15 mM HEPES buffer. During differentiation, the medium was refreshed every 2 days for 6 days. To induce functional maturation via air–liquid interface, the medium was added only to the lower compartment for an additional 4 days to expose the apical surface to air. Cells were used at passages previously validated to retain phenotypic stability after serial culture and post-thaw recovery. Only mycoplasma-negative hIEC inserts that achieved the prespecified TEER window (80–240 Ω·cm^2^) at day 14 were included in the assays. Batch-to-batch reproducibility was confirmed across ≥5 independent differentiation runs, which yielded consistent TEER and drug-response profiles^23^.

### Caco-2 cell culture and differentiation

Caco-2 cells were cultured following standard protocols to ensure reproducibility. Cells were maintained in Caco-2 culture medium composed of minimum essential medium (Thermo Fisher Scientific, no. 12571-071) supplemented with 10% FBS, 1% penicillin–streptomycin and 1% pyruvate (Gibco, no. 11360-070). The medium was refreshed every 2 days, and cells were passaged at 80–90% confluence using 0.25% trypsin–EDTA. For differentiation, Caco-2 cells were seeded on Transwell inserts at a density of 1.34 × 10^5^ cells/cm^2^ and cultured for 14 days. After seeding, the medium was replaced every 2 days with Caco-2 culture medium.

### TEER measurements

TEER was measured using an epithelial tissue volt/ohmmeter (EVOM2; WPI). Before beginning the assay, the electrodes were sterilized with 70% ethanol and washed with Dulbecco’s PBS (DPBS). After measuring TEER in cell-free inserts, the blank values were subtracted, and TEER was reported as Ω·cm^2^. TEER was recorded at 0, 24, 48 and 96 h after drug exposure (data not shown). On the basis of preliminary time course optimization, 96 h was selected as the standard endpoint because it reflected cumulative barrier disruption and aligned with the 3–5-day renewal cycle of the intestinal epithelium, representing the physiological turnover period of differentiated enterocytes. Dose–response curves were generated at concentrations of 0, 1, 5, 10, 50 and 100 µM to quantify the concentration-dependent barrier impairment across the drug panel^24^.

### Drug selection

In total, 17 drugs (from three pharmacological classes) known to cause adverse GI effects were selected on the basis of their clinical relevance, toxicity profiles and GI injury mechanisms. The panel included seven chemotherapeutic agents (paclitaxel, docetaxel, capecitabine, cyclophosphamide, cisplatin, 5-fluorouracil (5-FU) and doxorubicin) with well-known effects on rapidly dividing intestinal epithelial cells, five tyrosine kinase inhibitors (TKIs) (gefitinib, crizotinib, sunitinib, sorafenib and lapatinib, which can cause clinically severe diarrhea) and five nonsteroidal anti-inflammatory drugs (NSAIDs) (ibuprofen, diclofenac, naproxen, ketorolac and ketoprofen), which can cause mild GI mucosal injury.

### Drug treatment

After the terminal differentiation of hIECs and Caco-2 cells, drug-containing medium was added to the upper compartment, whereas drug-free medium was added to the lower compartment. After 48 h, the apical chamber received the same drug-containing medium, and the lower chamber received fresh drug-free medium. After 48 h (total drug exposure: 96 h), monolayers were processed for further analysis.

### Reverse transcription–quantitative polymerase chain reaction

Cells were washed twice with DPBS containing 0.1% diethyl pyrocarbonate (Sigma, no. D5758). The total RNA was extracted using an RNeasy Kit (Qiagen), and complementary DNA (cDNA) was synthesized from total RNA using the SuperScript IV cDNA Synthesis Kit (Invitrogen, no. 18090-050) according to the manufacturer’s instructions. Quantitative polymerase chain reaction was performed using a QuantStudio 5 thermal cycler (Thermo Fisher Scientific). Adult human small intestine RNA (Invitrogen, no. QS0626) was used as a positive control. Primer sequences are presented in Supplementary Table 1.

### Immunofluorescence analysis

Cells cultured on Transwell inserts were fixed with 4% paraformaldehyde (Biosesang, no. PC2031-100-00) for 5 min at room temperature and permeabilized using 0.2% Triton X-100 (Sigma, no. T9284) in PBS. After blocking with 4% bovine serum albumin (Bovogen, no. bsa100) for 1 h, the cells were incubated with primary antibodies overnight at 4 °C. Then, the cells were incubated with secondary antibodies for 1 h at room temperature in the dark and counterstained with Hoechst 33342 (Thermo Fisher Scientific, no. H3570) for 5 min. For vertical imaging of the polarized epithelium, the cells were cryoprotected by incubation with 30% sucrose for 48 h. Transwell membranes were vertically embedded in OCT compound (Saruka, no. HIO-0051) for subsequent cryosectioning. The sectioned samples were mounted onto glass slides, and the standard immunofluorescence protocol was followed as described above. Cells were observed under a confocal microscope (Zeiss, no. LSM800). The primary antibodies used in this study are listed in Supplementary Table 2.

### ATP cell viability assays

Cell viability assays were performed using the CellTiter-Glo Luminescent Cell Viability Assay kit (Promega, no. G7570) according to the manufacturer’s instructions. Cells were seeded into 96-well culture plates (Falcon, no. 353072) and treated with the indicated drugs for 96 h. After treatment, wells were washed once with DPBS, and 100 μL of fresh culture medium was added to each well. Then, an equal volume of CellTiter-Glo reagent was added to each well and incubated for 20 min at 37 °C. Luminescence was measured using a SpectraMax M3 microplate reader (Molecular Devices).

### Live–dead assays

For the live–dead staining analysis, we used a Cyto3D Live–Dead Assay Kit (TheWell Bioscience, no. BM01) according to the manufacturer’s instructions. The cells were washed once with Hank’s balanced salt solution (HBSS) containing calcium and magnesium (Gibco, no. 14025-092) and treated for 20 min with an HBSS working solution containing Cyto3D reagent diluted 1:50. The cells were imaged under a fluorescence microscope (Axio Observer 5; Zeiss), and the percentages of live (green) and dead (red) cells were quantified.

### ROS production assay

The reactive oxygen species (ROS) production was measured using the MitoSOX Red Mitochondrial Superoxide indicator (Invitrogen, no. M36008) according to the manufacturer’s instructions. In brief, after washing three times with HBSS containing calcium and magnesium (Gibco, no. 14025-092), the cells were incubated for 30 min with HBSS containing 500 nM MitoSOX Red reagent and imaged under a fluorescence microscope (IX51, Olympus). The fluorescence intensity was analyzed using ImageJ version 1.41o.

### Differential expression and KEGG pathway enrichment analyses

RNA sequencing (RNA-seq) was performed as described in our previous study^25^ using two independent biological replicates per condition. Batch effects were removed using the ComBat function from the sva package. Differentially expressed genes (DEGs) between control samples and samples treated with paclitaxel and docetaxel were identified using the Wilcoxon rank-sum test (two-sided *P* < 0.05) with a log_2_ fold-change ≥0.5.

The Kyoto Encyclopedia of Genes and Genomes (KEGG) pathway enrichment analysis was performed using the clusterProfiler package in R. The genes were mapped to Entrez Gene IDs, and the species was set to ‘*Homo sapiens*’. Pathways with a Benjamini–Hochberg adjusted *P* value (false discovery rate) <0.05 were considered significantly enriched.

### Pearson correlation analysis

To analyze linear correlations among assay performance (% reduction), GI toxicity scores and peak plasma concentrations (*C*_max_), Pearson correlation coefficients (*r*) were calculated using GraphPad Prism 10. The pairwise correlation heat map showed the strength and direction of the correlations (−1 ≤ *r* ≤ +1).

$$r=\frac{\sum ({x}_{{\rm{i}}}-\bar{x})({y}_{{\rm{i}}}-\bar{y})}{\sqrt{\sum {({x}_{{\rm{i}}}-\bar{x})}^{2}}\sqrt{\sum {({y}_{{\rm{i}}}-\bar{y})}^{2}}}.$$

### Calculation of the percent reduction of drug-induced cytotoxicity

Percent reduction was calculated to quantify drug-induced cytotoxicity, defined as the relative reduction in TEER or ATP cell viability compared with untreated controls, measured at the highest tested concentration (100 µM). The percent reduction was calculated as follows:

$$\% \hskip +1px\mathrm{Reduction}=\frac{(\mathrm{control}\,\mathrm{value}-\mathrm{value}\,\mathrm{at}\,100{\rm{\mu }}{\rm{m}})}{\mathrm{control}\,\mathrm{value}}\times 100.$$

For receiver operating characteristic (ROC) analyses, a unified threshold (≥50% TEER reduction) was applied to all assays to ensure a consistent interpretation of the results.

### Calculation of IC_15_ and IC_50_

Differentiated hIECs and Caco-2 cells were exposed to different concentrations of each drug (0, 1, 5, 10, 50 and 100 µM) for a total of 96 h. TEER and cell viability at 96 h were normalized to vehicle controls (100%). Concentration–response relationships were fitted to a four-parameter logistic model, and inhibitory concentration (IC_15_ and IC_50_) values were interpolated from the fitted curves. The four-parameter logistic equation is

$$Y=\mathrm{bottom}+\frac{(\mathrm{top}-\mathrm{bottom})}{(1+{10}^{(\log (\mathrm{ICvalue})-X)\times \mathrm{Hill}\,\mathrm{slope}}}.$$

IC_15_ and IC_50_ values reported as ‘>100 µM’ indicate right-censored data where no cytotoxicity or barrier disruption was detected at the highest tested concentration (100 µM). These cases were considered nontoxic within the assay range and were excluded from dose–response curve fitting.

### Calculation of the MOS

*C*_max_ values were compiled from peer-reviewed pharmacokinetic studies and US Food and Drug Administration (FDA) regulatory sources. When multiple reports were available for a given drug and dosing regimen, *C*_max_ was reported as median (IQR) and ranges (min–max) to two decimal places; when only a single report was available, IQR was reported as not available (NA). Reported arithmetic means from preliminary summaries were replaced, when possible, with steady-state medians. All *C*_max_ values were standardized to the specified dose and route and converted to a consistent unit. The margin of safety (MOS) compares an assay-derived inhibitory concentration with clinical exposure and was calculated as

$$\mathrm{MOS}=\mathrm{IC}\,\mathrm{value}/\mathrm{clinical}\,{C}_{\max }.$$

An MOS threshold of IC_15_:*C*_max_ <1 was interpreted as a potential clinical risk, that is, the therapeutic plasma concentration approaches or exceeds the concentration associated with a 15% decrement in assay values. IC_15_:*C*_max_ was prespecified as the primary, conservative early toxicity indicator; IC_50_:*C*_max_ was calculated secondarily for completeness.

### Calculation of total GI toxicity scores

We developed a quantitative GI toxicity scoring framework that combines severity and incidence. The total score for each drug was calculated as follows:

$$\mathrm{Total}\,\mathrm{GI}\,\mathrm{toxicity}\,\mathrm{score}=\mathrm{severity}\,\mathrm{grade}\,\mathrm{score}\times \mathrm{incidence}\,\mathrm{score}.$$

Common Terminology Criteria for Adverse Events (CTCAE) v5.0 severity grades (1–4) served as the basis for the severity component. Incidence was scored as 1 (uncommon), 2 (common) and 3 (very common). The total score ranged from 1 (grade 1, uncommon) to 12 (grade 4, very common), and risk was classified as low (≤3), moderate (4–8) or high (≥9). GI toxicity information (for example, diarrhea, mucositis, colitis, ileitis, perforation) was extracted from FDA labels and guidelines as well as curated sources (DrugBank, Drugs.com, Mayo Clinic) for short-course, fixed-dose regimens in patients without preexisting GI disease and was compared with model outcomes.

### Diagnostic accuracy and ROC analysis

Diagnostic accuracy metrics, including sensitivity, specificity, accuracy, positive predictive value and negative predictive value, were calculated by comparing predicted toxicity classifications (on the basis of predefined thresholds) against clinical toxicity (moderate/high risk versus low risk). Before constructing ROC curves, the threshold-dependent performance was evaluated across TEER percent reduction levels to determine the optimal cutoff. At each threshold, the diagnostic metrics were quantified as follows:

$$\mathrm{Youden}{\rm{\mbox{'}}}{\rm{s}}\,J=\mathrm{sensitivity}+\mathrm{specificity}-1,$$

$$\mathrm{Accuracy}=(\mathrm{TP}+\mathrm{TN})/(\mathrm{TP}+\mathrm{TN}+\mathrm{FP}+\mathrm{FN}),$$

where TP is true positive, TN is true negative, FP is false positive and FN is false negative. These metrics were used to identify performance plateaus and generate threshold plots (Supplementary Fig. 5a,b). ROC curves were constructed by plotting sensitivity (TP rate) against 1–specificity (FP rate). The area under the ROC curve (AUC) quantifies predictive performance, with values closer to 1 indicating higher predictive accuracy. ROC–AUC provided a threshold-independent summary of discrimination, whereas predefined thresholds (≥50% TEER reduction, MOS IC_15_:*C*_max_ <1 and MOS IC_50_:*C*_max_ <1) were selected for their clinical relevance in predicting GI toxicity. Thus, classification metrics at these thresholds reflected assay performance at decision points aligned with clinical use. AUCs were reported with 95% confidence intervals (CIs) calculated using the nonparametric DeLong method, and statistical significance (AUC >0.5) was assessed using the Mann–Whitney *U* test. Prespecified binary decision rules—at least 50% reduction in TEER or ATP at 100 µM; and IC_15_:*C*_max_ ratio of less than one—served as clinically useful thresholds for risk classification. All AUC values, 95% CIs and *P* values are shown in Supplementary Table 7.

### Statistical analysis

All statistical analyses were performed using Microsoft Excel, Python and GraphPad Prism version 10 (GraphPad Software). Pairwise comparisons were performed using Student’s *t*-test, and multiple groups were compared using one-way analysis of variance followed by the Holm–Sidak post hoc test, unless otherwise specified. Continuous variables were expressed as mean ± s.e.m. The CIs for AUCs were computed using standard error estimations on the basis of DeLong’s method, and the *P* values for ROC curves were calculated using the Mann–Whitney *U* test.

## Results

### hPS cell-derived hIECs mimic physiological features better than Caco-2 cells

**Fig. 1:**
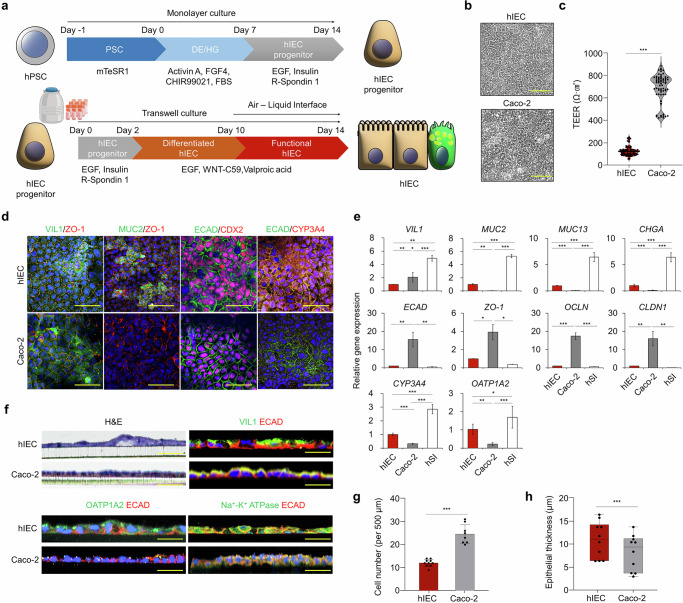
Characterization of hIEC and Caco-2 intestinal epithelium models. **a** A schematic illustration of the procedures followed to generate progenitor and functional hIECs. **b** The brightfield microscopy images showing the morphology of hIECs and Caco-2 cells. Scale bars, 100 μm. **c** TEER measurements indicating the barrier integrity of functional hIECs and Caco-2 cells. **d** The confocal immunofluorescence top-view images of hIEC and Caco-2 cell epithelial structures. Scale bars, 100 μm. **e** The relative expression levels of cell type and tight junction markers and drug metabolism-associated genes. **f** Cross-sectional confocal immunofluorescence images showing the vertical architecture of hIECs and Caco-2 cells. Scale bars, 100 μm. **g** A comparison of cell counts within the indicated regions. **h** Epithelial thicknesses of the hIEC and Caco-2 cell models. Images were acquired at 20× magnification. Data are presented as the mean ± s.e.m. (*n* > 4). Statistical analysis was performed using a *t*-test in **c**, **g** and **h** and by one-way analysis of variance in **e**. *P < 0.05, **P < 0.01, ***P < 0.001.

We first established intestinal epithelial models of drug-induced GI toxicity using hPS cell-derived hIECs and human enterocyte-like Caco-2 cells (Fig. 1a,b). As previously reported, both cell lines formed epithelial sheets with clear intercellular junctions, as observed under brightfield microscopy after differentiation (Fig. 1b). We compared barrier integrity by measuring TEER. Caco-2 cells exhibited much higher TEER (400–1,000 Ω·cm^2^) than hIECs (80 and 240 Ω·cm^2^) (Fig. 1c). Physiological TEER in the intestinal epithelium in vivo is 50–100 Ω·cm^2^ (ref. ^26^). To characterize cell type diversity and tight junctions in each model, we conducted immunostaining analyses for intestinal epithelial markers, including the enterocyte marker villin 1 (VIL1), the goblet cell marker mucin 2 (MUC2), the endoderm-specific marker homeobox protein CDX-2 (CDX2), the drug-metabolizing enzyme CYP3A4 (expressed in small intestine enterocytes) and the tight junction proteins zona occludens 1 (ZO-1) and E-cadherin (ECAD).

Both cell types expressed VIL1, CDX2, ECAD and ZO-1, whereas the goblet cell marker MUC2 was detected exclusively in hIECs (Fig. 1d). CYP3A4 was expressed in hIECs but not in Caco-2 cells originating from the large intestine (Fig. 1d). *VIL1* expression was significantly higher in Caco-2 cells than in hIECs (*P* = 0.004). By contrast, the expression of *MUC2* (*P* = 0.003), *MUC13* (*P* < 0.001) and the enteroendocrine cell marker chromogranin A (*CHGA*) (*P* < 0.001) was significantly higher in hIECs (Fig. 1e). The expression of the adherens junction marker *CDH1 (ECAD)* (*P* = 0.006) and other tight junction markers, including *ZO-1* (*P* = 0.022), *OCLN* (*P* < 0.001) and claudin 1 (*CLDN1*) (*P* = 0.003), was significantly higher in Caco-2 cells than in hIECs and human small intestine samples (Fig. 1e).

As the focus of this study was drug-induced GI toxicity, we compared the expression levels of genes involved in drug metabolism. *CYP3A4* expression was 3.15-fold higher in hIECs than in Caco-2 cells (*P* < 0.001) (Fig. 1e), consistent with the data presented in Fig. 1d. The organic anion-transporting polypeptide 1A2 gene (*OATP1A2*), responsible for the cellular uptake of chemotherapeutic drugs, was upregulated 4.66-fold in hIECs (*P* = 0.017) (Fig. 1e). The expression of 20 transporters and metabolic enzymes was substantially higher in hIECs than that previously reported in Caco-2 cells^16^. We confirmed the apical–basolateral polarization patterns of hIECs and Caco-2 cells using vertically sectioned epithelial sheets on a Transwell membrane. As expected, both cell types demonstrated polarized apical VIL1 expression. OATP1A2 was expressed in hIECs but not in Caco-2 cells (Fig. 1f), consistent with the results shown in Fig. 1e. The basolateral marker Na^+^/K^+^ ATPase was expressed in both cell types (Fig. 1f). Caco-2 cells exhibited higher densities (2.04 fold, *P* < 0.001) and a significantly lower epithelial thickness than hIECs (1.38 fold, *P* = 0.009), as evidenced by hematoxylin–eosin staining and immunostaining (Fig. 1g,h). Collectively, these findings suggest that the hIEC model closely resembles the small intestine and exhibits greater physiological relevance than Caco-2 cells (Table 1).

**Table 1:** Characterization of the hIEC and Caco-2 intestinal epithelial models.

Model	Mean TEER (Ω·cm² ± SEM)*	References TEER (Ω·cm²)	Barrier integrity	Origin	Cell composition
hIEC	129.6 ± 40.9	~80–240	Moderate integrity	hPS cell (in vitro)	Enterocyte, Paneth cell, goblet cell, enteroendocrine cell, stem cell
Caco-2	695.7 ± 126.1	~400–1,200	High integrity	Cancer cell line (in vitro)	Enterocyte
hSI	–	~50–100	Moderate integrity	(In vivo)	Enterocyte, Paneth cell, goblet cell, enteroendocrine cell, Tuft cell, stem cell, transit amplifying cell (epithelium)

TEER measurements (current study and reference values), barrier integrity levels, cellular origins and cell compositions are summarized.

### The TEER assay was more sensitive in detecting drug-induced GI toxicity than ATP cell viability assays

**Fig. 2:**
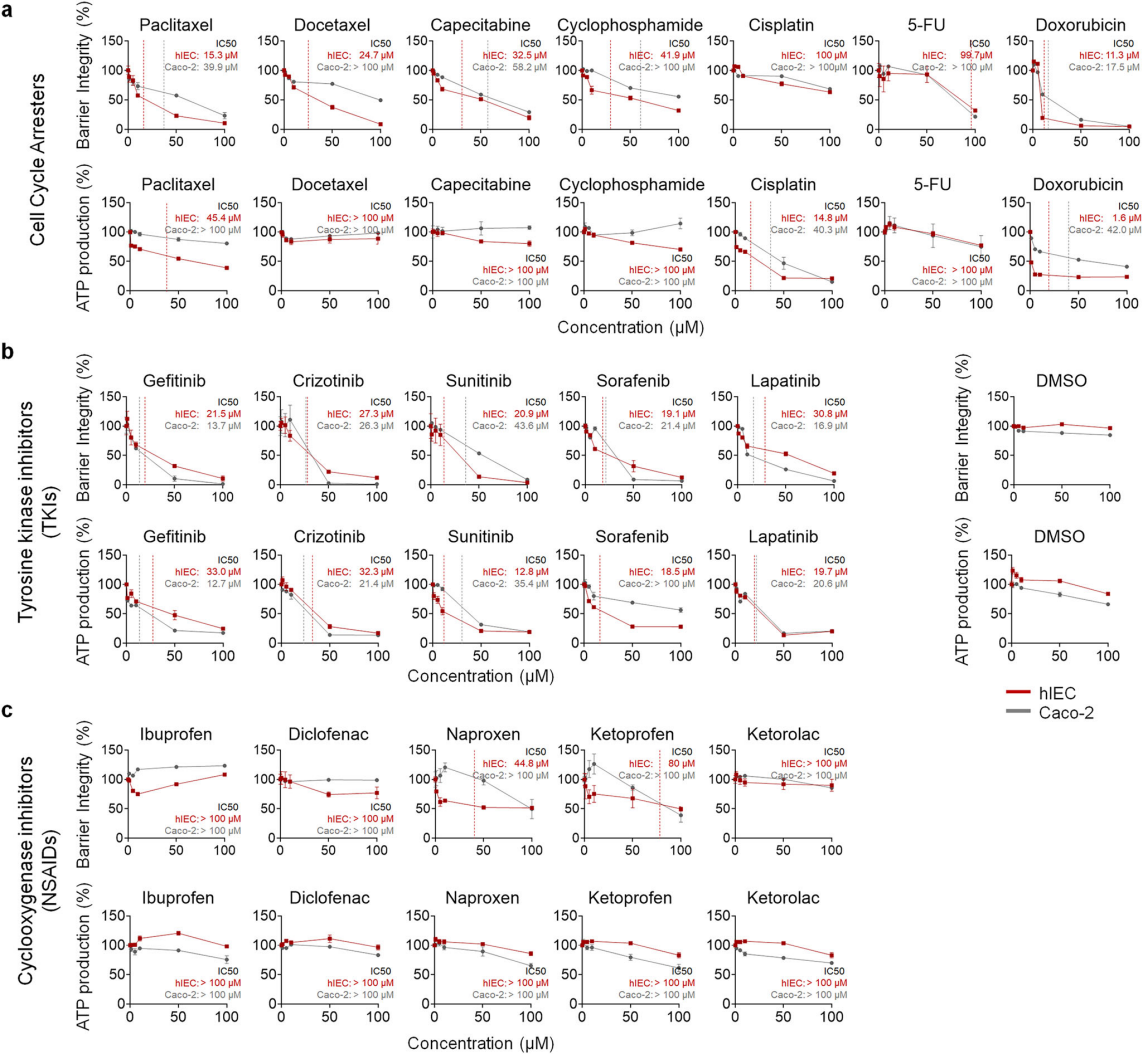
Comprehensive drug screening for cell cycle inhibitors, TKIs and NSAIDs using the hIEC and Caco-2 cell models. **a**–**c** Drug screening results for cell cycle inhibitors (**a**), TKIs (**b**) and NSAIDs (**c**) in the hIEC and Caco-2 cell intestinal epithelial models. The TEER assay results for each drug class are shown (top), and the cell viability assay results (at concentrations of 0, 1, 5, 10, 50 and 100 μM) are shown (bottom). The corresponding IC_50_ values are shown in red for hIECs and gray for Caco-2 cells. Data are presented as the mean ± s.e.m. of four independent experiments and are normalized to vehicle controls.

To compare the sensitivity of our TEER assay with conventional ATP cell viability assays, we screened 17 drugs, including seven cell cycle inhibitors (paclitaxel, docetaxel, capecitabine, cyclophosphamide, cisplatin, 5-FU and doxorubicin), five TKIs (gefitinib, crizotinib, sunitinib, sorafenib and lapatinib) and five NSAIDs (ibuprofen, diclofenac, naproxen, ketorolac and ketoprofen). The GI toxicity profile for each drug was classified by severity (grades 1–4) according to CTCAE v5.0. Toxicity levels are shown in Table 2 and Supplementary Table 3. Most drugs exhibited dose-dependent cytotoxicity, revealing a marked difference in sensitivity between the two assays, particularly for cell cycle inhibitors (Fig. 2 and Supplementary Fig. 1).

**Table 2:** Clinical GI toxicity profiles of the analyzed drugs.

Drug	Severity grade and score		Incidence and score		Total GI toxicity score (grade × incidence)	Clinical toxicity level
Paclitaxel	Grade 2	2	Very common	3	6	Moderate
Docetaxel	Grade 3	3	Very common	3	9	High
Capecitabine	Grade 3	3	Very common	3	9	High
Cyclophosphamide	Grade 2	2	Common	2	4	Moderate
Cisplatin	Grade 1	1	Very common	3	3	Low–moderate
5-FU	Grade 3	3	Very common	3	9	High
Doxorubicin	Grade 2	2	Common	2	4	Moderate
Gefitinib	Grade 2	2	Very common	3	6	Moderate
Crizotinib	Grade 2	2	Very common	3	6	Moderate
Sunitinib	Grade 2	2	Very common	3	6	Moderate
Sorafenib	Grade 2	2	Very common	3	6	Moderate
Lapatinib	Grade 3	3	Very common	3	9	High
Ibuprofen	Grade 1	1	Common	2	2	Low
Diclofenac	Grade ~1–2	1	Common	2	2	Low
Naproxen	Grade ~1–2	1	Common	2	2	Low
Ketoprofen	Grade ~1–2	1	Common	2	2	Low
Ketorolac	Grade 4	4	Uncommon	1	4	Moderate

GI toxicity levels (low, moderate and high) were determined using integrated severity grades and incidence categories, as described in the Methods. Clinical GI toxicity symptoms were based on FDA guidelines, the DrugBank Database, Drugs.com, information from the Mayo Clinic and relevant literature.

The ATP cell viability assay did not detect the cytotoxic effects of paclitaxel, docetaxel, capecitabine and cyclophosphamide even at the highest concentration tested (100 μM), whereas the TEER assay detected barrier disruption, especially in hIECs (Fig. 2a and Supplementary Fig. 1a). The TEER assay showed greater sensitivity in detecting the cytotoxicity of representative chemotherapeutics (5-FU and doxorubicin) than the viability assay, although cisplatin exhibited higher cytotoxicity in the latter (Fig. 2a and Supplementary Fig. 1a).

TKIs, which can inhibit tyrosine kinase receptors, such as the EGF receptor and platelet-derived growth factor receptor, showed severe cytotoxicity in both assays and cell types (Fig. 2b and Supplementary Fig. 1b), consistent with their clinical profiles (Table 2). NSAIDs exhibited low-to-moderate cytotoxicity, with the TEER assay being more sensitive than viability assays, particularly in hIECs (Fig. 2c and Supplementary Fig. 1c). The results of these assays are presented in Supplementary Table 4.

To validate the physiological relevance of our findings, we performed live–dead assays in the presence of cell cycle inhibitors. Paclitaxel, docetaxel, capecitabine and cyclophosphamide induced extensive cell death and barrier damage at 100 μM, which was consistent with the TEER results but not with the cell viability results (Fig. 2a and Supplementary Figs. 1a and 2). Cisplatin caused low-to-moderate cell death, in line with the TEER results; 5-FU and doxorubicin caused severe cell death, which drastically decreased TEER in both intestinal cell models (Fig. 2a and Supplementary Figs. 1a and 2). Collectively, these data show that the TEER assay using hIECs provides a more sensitive and physiologically relevant method for detecting drug-induced GI toxicity than ATP cell viability assays, particularly for cell cycle inhibitors, and demonstrates superior alignment with toxicity profiles.

### hIECs were better suited for assessing ROS-mediated cytotoxicity than Caco-2 cells

**Fig. 3:**
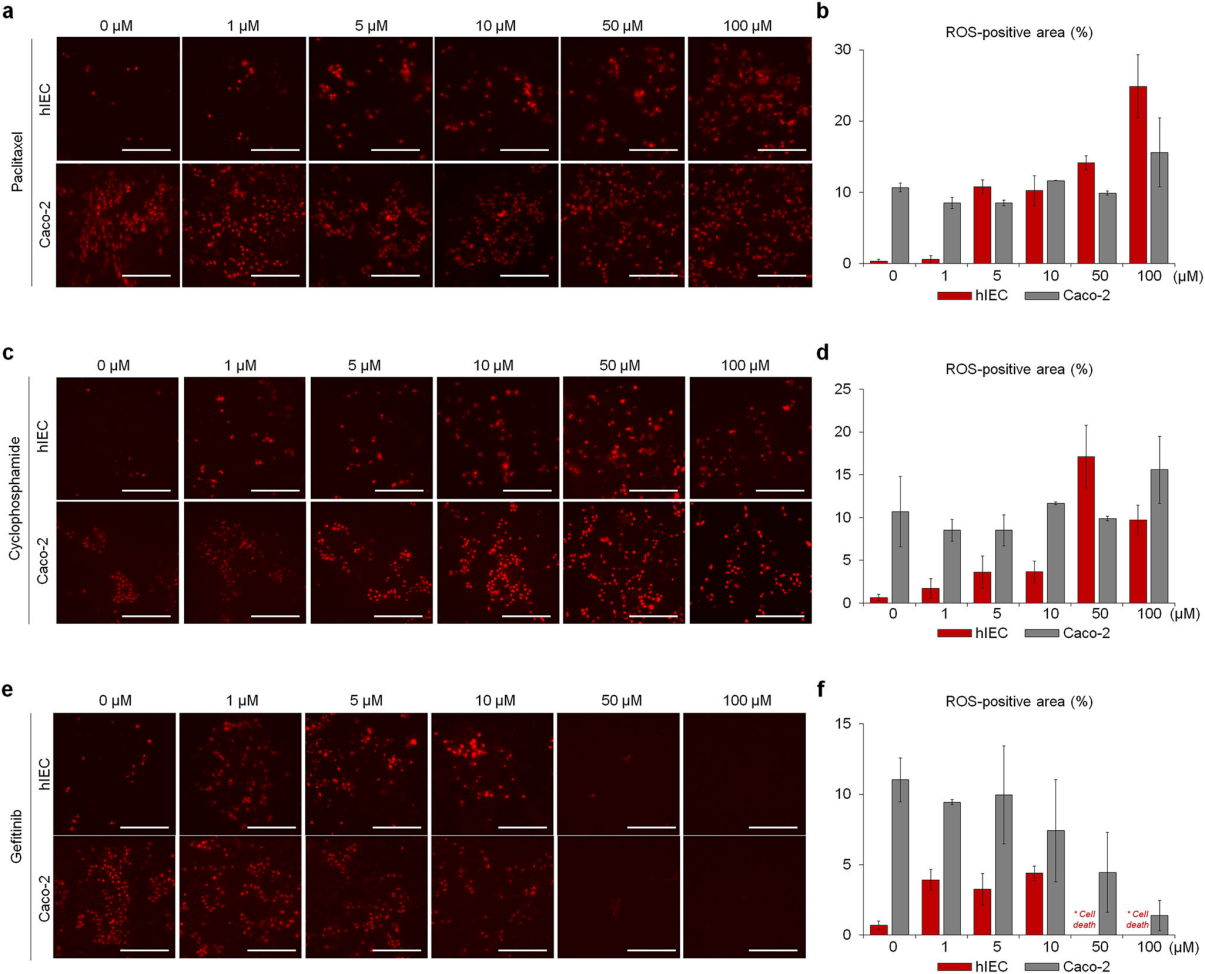
Dose-dependent changes in ROS production following drug treatment. **a**–**f** Representative fluorescence images and corresponding quantitative analyses of ROS-positive areas in hIECs and Caco-2 cells treated with paclitaxel (**a**, **b**), docetaxel (**c**, **d**) or gefitinib (**e**, **f**). **a**, **c**, **e**, Representative fluorescence images; **b**, **d**, **f**, quantitative analysis of ROS-positive areas. The ROS production was quantified in live cells using a mitochondria-specific fluorescent probe that reacts with superoxide anions. Data are presented as the mean ± s.e.m. from replicate experiments and are normalized to vehicle controls. **a**–**f** show the quantification of ROS-positive areas as a percentage of the total field area. The images were acquired at 20× magnification. Scale bar, 200 μm.

ROS, a mediator of chemotherapeutic toxicity, was measured in both cell types^27^. For dose–response analyses, we tested three drugs from distinct pharmacological classes—paclitaxel (microtubule stabilizer), cyclophosphamide (DNA damage inducer) and gefitinib (TKI). Caco-2 cells exhibited inherently high baseline ROS levels in the absence of the drug, limiting the ability to detect dose-dependent increases in ROS concentrations. By contrast, hIECs showed low baseline ROS levels with dose-dependent increases in ROS concentrations and the proportion of ROS-positive cells following exposure to paclitaxel or cyclophosphamide (Fig. 3a–d). Gefitinib increased ROS levels in hIECs up to 10 μM, followed by a decline at higher concentrations, consistent with severe mitochondrial injury and cell death (Fig. 3e,f and Supplementary Fig. 3). Collectively, these findings support the suitability of the hIEC model for assessing dose-dependent, ROS-mediated cytotoxicity and for predicting GI toxicity.

### Cytoskeleton-associated transcriptomic and KEGG pathway analysis in paclitaxel- and docetaxel-treated hIECs

**Fig. 4:**
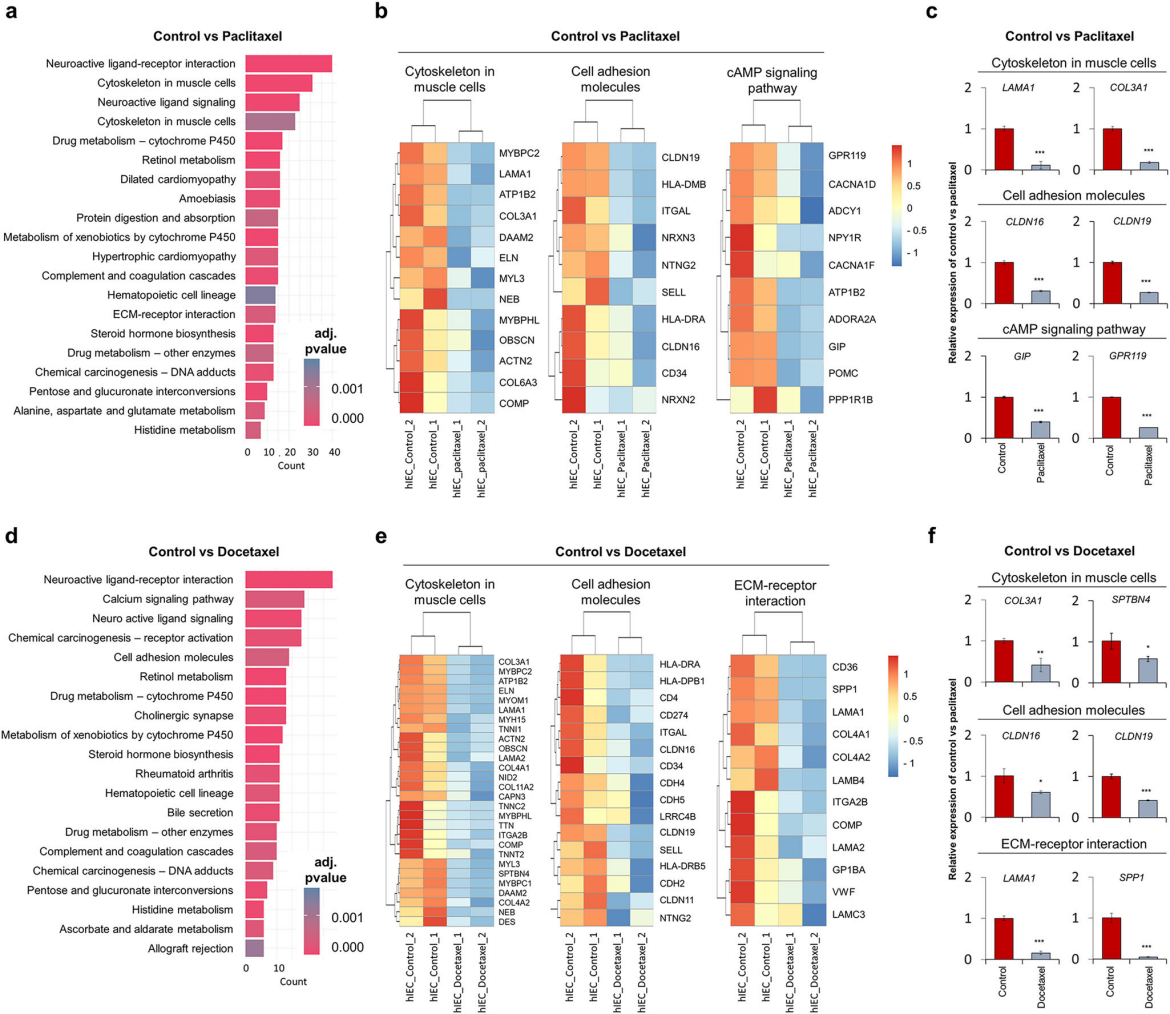
Cytoskeleton-related pathway alterations induced by paclitaxel and docetaxel in hIECs. **a** The KEGG pathway enrichment analysis of DEGs between control hIECs and paclitaxel-treated groups. The top enriched pathways are ranked by adjusted *P* value and gene count. **b** The heat maps showing expression profiles of representative DEGs associated with the cytoskeleton in muscle cells, cell adhesion molecules and the cAMP signaling pathway. **c** The qPCR validation of representative genes from the pathways shown in **b**. **d** The KEGG pathway enrichment analysis of DEGs between docetaxel-treated and control hIECs. **e** The heat maps showing expression profiles of representative DEGs associated with the cytoskeleton in muscle cells, cell adhesion molecules and ECM–receptor interaction. **f** The qPCR validation of selected representative genes from the pathways in **e**. Statistical significance was determined by conducting a *t*-test. Data are presented as mean ± s.e.m.; *P < 0.05, **P < 0.01, ***P < 0.001.

KEGG pathway enrichment analysis of downregulated DEGs in the paclitaxel group revealed the 20 most significantly enriched pathways (Fig. 4a). Among these, cytoskeleton in muscle cells (adjusted *P* = 0.0135), cell adhesion molecules (adjusted *P* = 0.00043) and cAMP signaling pathway (adjusted *P* = 0.0482) were prioritized for further investigation on the basis of their relevance to intestinal barrier integrity and statistical significance^17,19^. Heat maps showed that the expression profiles of these pathways differed between the paclitaxel and control groups (Fig. 4b). Quantitative PCR (qPCR) analysis indicated that representative genes (*LAMA1*, *COL3A1*, *CLDN16*, *CLDN19*, *GIP* and *GPR119*) were markedly downregulated, in line with the RNA-seq results (Fig. 4c).

In the docetaxel group, the analysis of downregulated DEGs similarly identified enrichment in cytoskeleton in muscle cells (adjusted *P* = 1.05 × 10^−8^), cell adhesion molecules (adjusted *P* = 0.00113) and extracellular matrix (ECM)–receptor interaction (adjusted *P* = 1.85 × 10^−5^) (Fig. 4d). The heat maps showed that the expression profiles of these pathways differed between the paclitaxel and docetaxel groups (Fig. 4e). The qPCR analysis showed that key genes (*SPTBN4*, *COL3A1*, *CLDN16*, *CLDN19*, *LAMA1* and *SPP1*) were downregulated, corroborating the transcriptomic results (Fig. 4f).

A 3D multidimensional-scaling plot of downregulated DEGs showed a clear separation of the paclitaxel- and docetaxel-treated groups from controls along dimension 2, indicating distinct transcriptional responses to both taxanes (Supplementary Fig. 4a). The corresponding heat map, (Supplementary Fig. 4b), which focused on the cAMP signaling pathway and regulation of the actin cytoskeleton, showed higher expression in controls and lower expression in drug-treated samples, consistent with pathway-level downregulation^17,19^. The qPCR analysis demonstrated that representative genes from these pathways (*ATP1B2*, *GPR119*, *ADORA2*, *CACNA1D*, *POMC* and *FGF9*) were downregulated, in line with the transcriptomic results and heat map (Supplementary Fig. 4c). In line with the transcriptomic enrichment of cytoskeleton- and adhesion-related pathways (Fig. 4), the qPCR analysis also showed that paclitaxel and docetaxel downregulated *RHOA*, *ROCK2*, *CDC42*, *ACTG1*, *ACTN1* and *ELN* (Supplementary Fig. 4d). These results indicate that the taxane-induced suppression of Rho-GTPase and actin-junction–ECM gene networks underlies the observed decline in barrier integrity.

### The hIEC-based TEER assay reliably predicts GI toxicity

We compared the predictive performance of the hIEC-based TEER assay, a clinically relevant method to detect GI toxicity, with that of the Caco-2-based TEER assay and ATP cell viability assays in both cell types. Drugs were classified as toxic or nontoxic using two criteria: MOS threshold (IC_15_:*C*_max_ < 1) and percent reduction threshold (≥50% decrease in TEER or ATP viability at 100 µm). The ≥50% TEER reduction cutoff was supported by threshold performance analysis (Supplementary Fig. 5a,b), which showed a broad plateau of high Youden’s *J* (≈0.92) and accuracy (≈0.94) for the hIEC TEER model at ~50–65%, whereas the Caco-2 TEER model attained a lower plateau (*J* ≈ 0.75) at ≥60%. *C*_max_ values are summarized as median (IQR) with min–max for each drug, and the corresponding MOS metrics (median and (min–max)) are presented in Supplementary Table 5 to show exposure variability across clinical conditions. The incidence of severe GI toxicity grade ≥3 for the 17-drug panel is summarized in Supplementary Table 6. The hIEC-based TEER assay recapitulated the clinically observed range of GI toxicity levels across and within pharmacological classes (Supplementary Table 6). The IC_15_, IC_50_ and percent reduction values derived from both assays in hIECs and Caco-2 cells are summarized in Table 3.

**Table 3:** *C*_max_, IC_15_, IC_50_ and percent reduction values obtained from intestinal toxicity assays.

Drug	Clinical *C*_max_ (μM)	IC_15_ (μM)				IC_50_ (μM)				Reduction (%)			
		hIEC TEER	Caco-2 TEER	hIEC ATP	Caco-2 ATP	hIEC TEER	Caco-2 TEER	hIEC ATP	Caco-2 ATP	hIEC TEER	Caco-2 TEER	hIEC ATP	Caco-2 ATP
Paclitaxel	5.1	2.71	7.04	8.01	69.00	15.33	39.91	45.41	391.00	89.51	76.32	60.91	19.38
Docetaxel	5.1	4.36	19.25	88.94	291.18	24.69	109.10	504.00	1650.00	91.28	50.38	10.69	1.71
Capecitabine	13.7	5.74	10.27	62.59	>100 μм	32.52	58.21	354.70	>100 μм	79.92	70.45	18.49	−10.35
Cyclophosphamide	122.0	7.40	22.84	40.50	>100 μм	41.91	129.40	229.50	>100 μм	67.94	44.51	29.32	−15.13
Cisplatin	14.8	30.49	44.45	2.62	7.10	172.80	251.90	14.84	40.26	36.84	31.31	78.90	84.85
5-FU	34.1	19.31	17.60	87.78	74.47	109.40	99.74	497.40	422.00	65.48	78.64	22.64	24.14
Doxorubicin	7.6	1.99	3.08	0.28	7.42	11.28	17.48	1.61	42.04	95.35	94.75	76.28	58.92
Gefitinib	0.5	3.80	2.41	5.81	2.24	21.51	13.68	32.95	12.72	90.44	98.16	75.25	82.42
Crizotinib	1.0	4.81	4.64	5.70	3.78	27.25	26.31	32.32	21.41	88.04	98.27	82.94	86.50
Sunitinib	0.3	3.68	7.70	2.25	6.24	20.87	43.61	12.77	35.35	96.79	91.17	80.96	80.57
Sorafenib	6.5	3.36	3.77	3.26	19.82	19.05	21.38	19.32	112.30	87.72	93.32	75.98	43.81
Lapatinib	4.2	5.44	2.98	3.48	3.64	30.82	16.89	19.73	20.63	80.26	93.56	79.81	79.31
Ibuprofen	145.0	>100 μм*	>100 μм	>100 μм	59.49	>100 μм	>100 μм	>100 μм	337.10	−8.14	−23.28	1.75	24.28
Diclofenac	5.0	43.52	1018.24	>100 μм	110.47	246.60	5,770.00	>100 μм	626.00	23.83	0.99	3.31	16.63
Naproxen	313.0	7.90	37.01	177.71	42.25	44.75	209.70	1,007.00	239.40	47.84	54.68	14.01	34.81
Ketoprofen	10.8	14.11	25.50	150.74	30.46	79.98	144.50	854.20	172.60	49.84	59.11	17.00	38.82
Ketorolac	11.0	134.29	156.05	150.74	34.82	761.00	884.30	854.20	197.30	10.60	14.63	17.00	30.03

*C*_max_ values (µM) were obtained from referenced sources. IC_15_, IC_50_ and percent reduction metrics were measured using TEER and ATP cell viability assays in hIEC and Caco-2 intestinal epithelial models. IC values reported as ‘>100 µM’ indicate right-censored data where no cytotoxicity or barrier disruption was detected at the highest tested concentration (100 µM). These cases were considered nontoxic within the assay range and were excluded from dose–response curve fitting.

*IC_15_ and IC_50_ values reported as ‘>100 µM’ indicate right-censored data where no cytotoxicity or barrier disruption was detected at the highest tested concentration (100 µM). These cases were considered nontoxic within the assay range and were excluded from curve fitting.

**Fig. 5:**
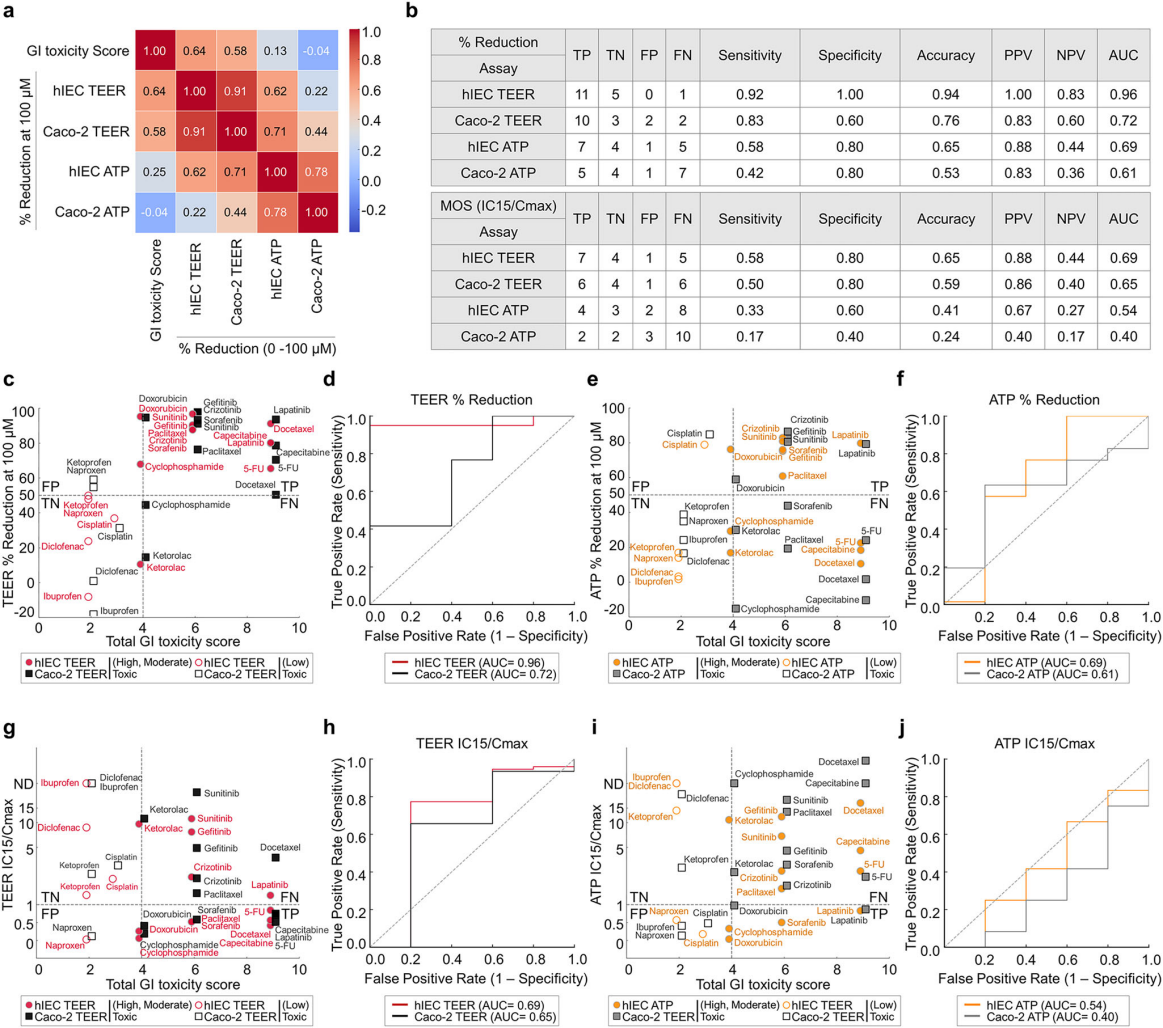
Diagnostic accuracy and clinical relevance of intestinal toxicity assays. **a** A heat map showing Pearson correlation coefficients between the GI toxicity scores of the indicated drugs (tested at 100 µm) and the corresponding assay-derived percent reductions (TEER and ATP cell viability assays). **b** Diagnostic accuracy metrics (sensitivity, specificity, accuracy, positive predictive value, negative predictive value and ROC–AUC) were calculated using ≥50% TEER reduction and MOS (IC_15_:*C*_max_ <1). **c** A scatter plot distinguishing TPs, TNs, FPs and FNs on the basis of ≥50% TEER reduction and GI toxicity scores (threshold of 4). hIECs and Caco-2 cells are represented with red circles and black squares, respectively; filled symbols indicate high/moderate toxicity, and empty symbols indicate low toxicity. **d** ROC curves for TEER assay accuracy using ≥50% reduction in TEER as a criterion. **e** Quadrant scatter plots (ATP cell viability assay, ≥50% reduction) that distinguished TPs, TNs, FPs and FNs on the basis of GI toxicity scores (threshold of 4). The symbols are described in **c**, hIECs are shown in orange circles and Caco-2 cells are shown in gray squares. **f** ROC curves for assessing the accuracy of call viability assays using ≥50% reduction. **g** A scatter plot (TEER assay, IC_15_:*C*_max_ <1) distinguishing TPs, TNs, FPs and FNs based on GI toxicity scores (threshold of 4). The symbols are described in **c**. **h** ROC curves for assessing TEER assay accuracy on the basis of IC_15_:*C*_max_ <1. **i** A scatter plot (cell viability assay, IC_15_:*C*_max_ <1) showing the distinction between TPs, TNs, FPs and FNs on the basis of GI toxicity scores (threshold of 4). The symbols are described in **e**. **j** ROC curves for cell viability assay accuracy on the basis of IC_15_:*C*_max_ <1. Data are expressed as mean ± s.e.m. of four independent experiments. The statistical significance of ROC analysis was assessed using the Mann–Whitney *U* test, and 95% CIs were computed using the nonparametric DeLong method. Panel-specific AUC (95% CI, DeLong) and *P* values (Mann–Whitney *U*) were as follows: for **d**, hIEC TEER (≥50%): 0.96 (0.82–1.00), *P* < 0.001; Caco-2 TEER (≥50%): 0.72 (0.48–0.89), *P* = 0.036. For **f**, hIEC ATP (≥50%): 0.69 (0.44–0.88), *P* = 0.049; Caco-2 ATP (≥50%): 0.61 (0.37–0.81), *P* = 0.092 (n.s.). For **h**, hIEC TEER (MOS IC15:*C*_max _<1): 0.69 (0.43–0.90), *P* = 0.042; Caco-2 TEER (MOS): 0.65 (0.40–0.85), *P* = 0.059 (n.s.). For **j**, hIEC ATP (MOS): 0.54 (0.32–0.74), *P* = 0.214 (n.s.); Caco-2 ATP (MOS): 0.40 (0.22–0.58), *P* = 0.308 (n.s.). n.s., not significant. Complete statistics are presented in Supplementary Table 7.

The total GI toxicity scores showed moderate correlations with percent reduction (Pearson’s *r* = 0.64 for the hIEC-based TEER assay; *r* = 0.58 for the Caco-2-cell-based TEER assay; Fig. 5a). By contrast, the results of ATP cell viability assays correlated weakly or negligibly with GI toxicity scores (*r* = 0.25 for hIECs; *r* = −0.04 for Caco-2 cells; Fig. 5a). Consistent with threshold plots (Supplementary Fig. 5a,b), the ≥50% TEER reduction cutoff lay within the early portion of the performance plateau (~50–65%) for hIECs, aligning with the physiological onset of barrier loss rather than late cytotoxicity. These findings indicate that the hIEC-based TEER assay detects clinically relevant intestinal barrier disruption rather than general cytotoxicity, as measured in ATP cell viability assays, demonstrating the utility of TEER assays for the early detection of intestinal barrier disruption^26,28^.

Guided by performance plateaus (Supplementary Fig. 5a,b), toxicity was classified on the basis of the ≥50% TEER reduction cutoff (Fig. 5b,c,e,g,i and Supplementary Table 7). All tested drugs were classified as low risk or moderate/high risk on the basis of a validated GI toxicity scoring framework (threshold ≥4; Table 2 and Supplementary Table 3). Scatter plots enabled the visual distinction of TPs, FNs, TNs and FPs for each assay using percent reduction and MOS (Fig. 5b,c,e,g,i). Using the percent reduction criterion, the hIEC-based TEER assay achieved superior diagnostic performance, correctly classifying 11 of 12 toxic drugs (paclitaxel, docetaxel, capecitabine, cyclophosphamide, 5-FU, doxorubicin, gefitinib, crizotinib, sunitinib, sorafenib, lapatinib; sensitivity 92%) and all five low-toxicity drugs (cisplatin, ibuprofen, diclofenac, naproxen, ketoprofen; specificity 100%), resulting in an overall accuracy of 94% (Fig. 5b,c and Supplementary Table 7). Conversely, the Caco-2-based TEER assay had lower sensitivity (83%), specificity (60%) and accuracy (76%), notably misclassifying cyclophosphamide and ketorolac (Fig. 5b,c and Supplementary Table 7). Both ATP cell viability assays exhibited markedly lower predictive accuracy (approximately 65%), primarily owing to low sensitivity (~58%), despite moderate specificity (80%) (Fig. 5b,e and Supplementary Table 7). Overall, these classification patterns (Supplementary Table 7) indicate that the hIEC TEER assay had the smallest number of FP and FN classifications and the highest predictive accuracy, whereas the Caco-2 TEER and cell viability assays exhibited progressively higher FN rates and lower accuracy. Moreover, the paired dot plot in Supplementary Fig. 5c parallels the quadrant separation in Fig. 5c, showing larger and more consistent TEER reductions in hIECs among toxic agents while preserving specificity for low-toxicity drugs. Ketorolac was identified as nontoxic (FN) using both TEER assays, reflecting the limitations of these assays for detecting specific GI toxicity mechanisms, such as chemical irritation or prostaglandin inhibition, highlighting the need for complementary assays (Fig. 5c,e and Supplementary Table 7). The ROC curve analysis showed that the hIEC-based TEER assay using the ≥50% reduction threshold achieved the highest diagnostic accuracy (AUC of 0.96), outperforming the Caco-2 TEER assay (AUC of 0.72) and both cell viability assays (AUC ≤0.69) (Fig. 5d,f; 95% CIs and Mann–Whitney *P* values are reported in Supplementary Table 7 and summarized in the legend of Fig. 5). Among toxic compounds, cyclophosphamide was detected only in the hIEC-based TEER assay (TP), whereas the Caco-2 TEER assay yielded FN, consistent with chemotherapy-induced mucositis and tight junction disruption (Table 2 and Supplementary Table 7). By contrast, two NSAIDs—naproxen and ketoprofen—appeared as FPs in the Caco-2 TEER assay, reflecting acute prostaglandin-dependent permeability changes rather than true barrier disruption.

The MOS (IC_15_:*C*_max_) threshold of 1 was selected as a scientifically robust and clinically meaningful criterion, representing the precise boundary where the minimal drug concentration causing early intestinal epithelial damage (IC_15_) aligns with clinically achievable drug concentrations (*C*_max_). Using this MOS threshold, the hIEC-based TEER assay achieved relatively higher sensitivity (58%), specificity (80%) and accuracy (65%) than the other assays. However, the overall accuracy of the MOS threshold was lower than that of the ≥50% reduction threshold (Fig. 5g,i and Supplementary Table 7). This discrepancy highlights the inherent limitations of systemic exposure-based thresholds, which may underestimate local mucosal toxicity, particularly for orally administered drugs (for example, gefitinib, crizotinib, sunitinib and lapatinib), resulting in FNs (Supplementary Table 7). Moreover, MOS analyses based on IC_15_:*C*_max_ <1 demonstrated substantially lower predictive accuracy across all assays, confirming that IC_50_ values frequently exceed clinically achievable drug concentrations, thus limiting their predictive utility (Supplementary Table 8).

Collectively, these findings underscore the robust predictive performance of the hIEC-based TEER assay, particularly when the percent reduction criterion is applied. This criterion, supported by previous experimental findings highlighting the early detection of barrier disruption at biologically relevant concentrations^29–32^, provides a highly sensitive method for assessing drug-induced GI toxicity.

## Discussion

Our findings established the hIEC-based TEER assay as a highly effective and clinically relevant platform for assessing drug-induced GI toxicity, effectively addressing the critical limitations of conventional methods. Unlike widely used ATP cell viability assays or the Caco-2 TEER assay, our hIEC model uses nontransformed normal hPS cell-derived hIECs to enhance physiological relevance^16^. This crucial difference was evident in the higher sensitivity and specificity of our assay for detecting intestinal barrier disruption caused by diverse classes of pharmacological inhibitors, including cell cycle inhibitors, TKIs and NSAIDs.

The hIEC-based TEER assay demonstrated high predictive accuracy, accurately identifying drugs associated with GI toxicity using rigorous criteria, including symptom severity scores and incidence rates. Our comprehensive scoring framework based on CTCAE v5.0 criteria substantially enhanced the translational validity of in vitro findings, enabling precise stratification of the tested drugs into clinically meaningful categories based on toxicity levels (low, moderate or high). IC_50_ values frequently exceed clinically achievable drug concentrations^33^, limiting their predictive accuracy. Moreover, relying solely on *C*_max_ may underestimate intestinal mucosal exposure. Therefore, assays using high drug concentrations (for example, 100 µM) are critical for the early assessment of toxicity, whereas MOS analyses relate assay potency to clinical exposure. Integrating mucosal exposure data, drug formulation characteristics and pharmacokinetic profiles is recommended for a comprehensive assessment of GI toxicity. To address these gaps, we validated an alternative, physiologically meaningful threshold (≥50% TEER reduction at 100 µm), which markedly increased the clinical utility of the hIEC TEER assay. This criterion detected marked epithelial barrier disruption, reflecting clinically relevant GI damage. Consistent with this rationale, we observed a broad plateau in performance in the hIEC TEER model (Youden’s *J* ≈ 0.92; accuracy of approximately 0.94) across the TEER reduction range of 50–65% (Supplementary Fig. 5a,b), supporting the use of a ≥50% reduction cutoff for the early detection of barrier disruption. Together, these complementary metrics strengthen the translational assessment of GI safety.

The predictive accuracy of the hIEC-based TEER assay using the percent reduction criterion was high, demonstrating strong accuracy for orally administered drugs (TKIs and NSAIDs) directly exposed to the intestinal mucosa, as well as for intravenously administered anticancer drugs (cell cycle inhibitors). In addition, the assay accurately predicted GI toxicity for all tested drugs, except for ketorolac (FN), reflecting its broader predictive applicability despite the need for complementary assays to detect alternative GI toxicity mechanisms, such as chemical irritation or prostaglandin inhibition. The clinical advantage of the hIEC-based TEER assay was confirmed by percent reduction analysis, demonstrating that ≥50% TEER reduction at 100 µM—well above *C*_max_—differentiated between safe and toxic drug exposure levels. Importantly, the hIEC-based TEER assay discriminated between GI toxicity levels across and within pharmacological classes (Supplementary Table 6).

An MOS (IC_15_:*C*_max_) threshold of 1 was chosen as a clinically meaningful and scientifically robust criterion, representing the boundary at which the lowest concentration causing early intestinal epithelial damage (IC_15_)^32^ coincides with clinically achievable drug concentrations (*C*_max_). This threshold serves as a sensitive indicator of early GI toxicity, directly linking TEER assay results to therapeutic exposure. Given the observed sensitivity and predictive accuracy of the hIEC-based TEER assay, this MOS threshold is appropriate for conservative, biologically relevant early risk assessments, particularly in cases of chronic or repeated drug administration. Accordingly, ≥50% TEER reduction served as the primary decision criterion, and MOS (IC_15_:*C*_max_ <1) was used to aid interpretation of systemic exposure, thereby reinforcing the role of barrier function measurements as the most reliable indicators of GI toxicity. ROC metrics support this hierarchy (Fig. 5 and Supplementary Tables 7 and 8). Furthermore, measurable disruptions in intestinal barrier integrity often precede overt cytotoxic effects detected in ATP-based viability assays^34–36^, supporting TEER-based barrier integrity monitoring as an early, sensitive indicator of GI injury. This approach prioritizes barrier function as a predictive marker of GI manifestations, including diarrhea and mucositis. Recent advances, such as the RepliGut planar model derived from adult intestinal stem cells^37^, emphasize the importance of patient-specific intestinal epithelial platforms for predicting drug toxicity. However, adult tissue-derived models inherently reflect donor variability, potentially limiting generalizability. By contrast, our hPS cell-derived hIEC platform provides a scalable and reproducible system that performs consistently under standardized culture and assay conditions, thereby improving translational relevance across diverse patient populations.

Bulk RNA-seq complemented functional assays to identify the mechanisms underlying barrier disruption. The results showed a convergent weakness across intestinal structural integrity networks, including cytoskeletal, junctional and ECM–receptor pathways. We observed the downregulation of ECM–receptor interactions and cAMP signaling, consistent with cytoskeletal disruption^17,20,21,38^. These patterns suggest that compromised structural integrity is associated with impaired epithelial function and homeostasis. The same directional changes were observed for paclitaxel and docetaxel, indicating a convergent molecular response to microtubule-targeting agents. In parallel, gene sets essential for intestinal function, including those involved in drug metabolism, were markedly downregulated, consistent with a cascade of dysfunction. The concurrent downregulation of genes associated with canonical cytoskeletal and ECM components (for example, *LAMA1*, *COL3A1*, *CLDN16*, *CLDN19*, *GIP*, *GPR119*) suggests that chemotherapeutic toxicity compromises epithelial architecture and the biochemical interactions that sustain barrier function and homeostasis. Consistent with these results, studies on cellular aging found significant associations between cytoskeletal destabilization, metabolic dysfunction and diminished regenerative capacity^39–41^. Collectively, these findings indicate that the cytoskeleton underlies the link between chemotherapeutic exposure and intestinal epithelial injury.

Our results show that measurable intestinal barrier disruption often precedes overt cytotoxicity determined by ATP cell viability assays^35,36,42^. Unlike destructive cell viability and cytotoxicity assays conducted at the selected endpoint, TEER measurements are noninvasive and reproducible^32^. For instance, ATP cell viability assays can be temporarily confounded by early increases in ROS and concomitant elevations in ATP and NADH production that occur as a compensatory metabolic response before cell death. Such transient elevations are probably driven by mitochondrial overactivation, as evidenced by the dose-dependent increase in ROS (Fig. 3). Metabolic activation may mask cytotoxicity, which explains the low detection of ROS in viability assays (Fig. 2), particularly with cell cycle inhibitors, despite evidence of cell death. By directly quantifying paracellular ion permeability, TEER serves as a sensitive and functionally relevant early indicator of intestinal barrier impairment. This finding marks a fundamental paradigm shift in preclinical toxicity screening, prioritizing barrier function as a pivotal, predictive marker of GI manifestations, including diarrhea, mucositis and severe complications. The hPS cell-derived hIEC model strengthens these findings by providing an epithelial model that more closely mimics human intestinal structure and function. Directed differentiation on monolayer cultures containing diverse epithelial cell types provides bidirectional (apical–basolateral) access and enables the real-time assessment of barrier function, thereby strengthening the assay’s translational utility. Therefore, the hIEC model is well-suited for GI toxicity screening and mechanistic studies, particularly for ROS-driven cytotoxicity induced by cell cycle inhibitors^27^. By contrast, elevated baseline ROS in Caco-2 cells limits sensitivity to drug-induced increases in ROS, whereas hIECs reliably detect dose-dependent increases in ROS, supporting detailed investigation of chemotherapy-related GI toxicity^34,43–45^. The hIEC-based TEER assay reliably distinguishes highly toxic chemotherapeutics and TKIs from low-toxicity drugs, enabling the early detection of GI toxicity, reducing clinical failure rates and improving drug development efficiency^6,16,46,47^.

This study has limitations. First, although the drug screening panel is comprehensive, it does not encompass the full spectrum of drug classes currently in clinical use, potentially limiting generalizability. Second, we prioritized the assessment of acute toxicity over chronic toxicity, which is particularly relevant for long-term medications such as NSAIDs^48^. Third, experiments were conducted using a conventional intestinal model, without accounting for inflammatory or disease-specific contexts that may influence GI toxicity in clinical settings. Future studies should incorporate repeated dosing and greater physiological complexity, including the addition of nonepithelial cells, such as immune cells, to improve the physiological relevance of this assay^4,49^. Fourth, the sample size was limited; thus, the validation of the hIEC-based TEER system using a broader panel of compounds is warranted.

By benchmarking our hIEC-based TEER assay system against conventional methodologies and correlating its findings with clinical data, we demonstrated its enhanced physiological relevance for clinical translation and the precision of drug-induced GI toxicity assessments. This model accurately recapitulates intestinal epithelial responses, closely mimics in vivo conditions and provides a reliable and informative platform for the early screening of drug toxicity. Because of its improved predictive accuracy, cost-effectiveness and operational efficiency, this system holds substantial promise for performing GI safety assessments, ultimately helping to reduce adverse GI events and improve risk management.

## Supplementary information

Supplementary Information

Supplementary Table 4

## Data Availability

The data that support the findings of this study are available from the corresponding author upon reasonable request.

## References

[1] Adjei, A. A. A review of the pharmacology and clinical activity of new chemotherapy agents for the treatment of colorectal cancer. *Br. J. Clin. Pharmacol.* **48**, 265–277 (1999). 10510136 10.1046/j.1365-2125.1999.00010.x PMC2014335

[2] Workman, M. D. G. A. P. Discovering novel chemotherapeutic drugs for the third millenium. *Eur. J. Cancer* **35**, 2010–2030 (1999). 10711243 10.1016/s0959-8049(99)00280-4

[3] Akbarali, H. I., Muchhala, K. H., Jessup, D. K. & Cheatham, S. Chemotherapy induced gastrointestinal toxicities. *Adv. Cancer Res.* **155**, 131–166 (2022). 35779873 10.1016/bs.acr.2022.02.007 PMC10033220

[4] Hijos-Mallada, G., Sostres, C. & Gomollón, F. NSAIDs, gastrointestinal toxicity and inflammatory bowel disease. *Gastroenterol. Hepatol.* **45**, 215–222 (2022). 34157367 10.1016/j.gastrohep.2021.06.003

[5] Dahlgren, D. & Lennernas, H. Review on the effect of chemotherapy on the intestinal barrier: epithelial permeability, mucus and bacterial translocation. *Biomed. Pharmacother.* **162**, 114644 (2023). 37018992 10.1016/j.biopha.2023.114644

[6] Liu, J. et al. Mechanism and treatment of diarrhea associated with tyrosine kinase inhibitors. *Heliyon* **10**, e27531 (2024). 38501021 10.1016/j.heliyon.2024.e27531 PMC10945189

[7] Rodrigues, D. et al. Drug-induced gene expression profile changes in relation to intestinal toxicity: state-of-the-art and new approaches. *Cancer Treat. Rev.* **77**, 57–66 (2019). 31279169 10.1016/j.ctrv.2019.06.004

[8] Belair, D. G. et al. Human ileal organoid model recapitulates clinical incidence of diarrhea associated with small molecule drugs. *Toxicol. In Vitro* **68**, 104928 (2020). 32622998 10.1016/j.tiv.2020.104928

[9] Rodrigues, D. et al. New insights into the mechanisms underlying 5-fluorouracil-induced intestinal toxicity based on transcriptomic and metabolomic responses in human intestinal organoids. *Arch. Toxicol.* **95**, 2691–2718 (2021). 34151400 10.1007/s00204-021-03092-2 PMC8298376

[10] Rodrigues, D. et al. Unravelling mechanisms of doxorubicin-induced toxicity in 3D human intestinal organoids. *Int. J. Mol. Sci.* **23**, 1286 (2022). 10.3390/ijms23031286 PMC8836276 35163210

[11] Ghosh, S. S., Wang, J., Yannie, P. J. & Ghosh, S. Intestinal barrier dysfunction, LPS translocation, and disease development. *J. Endocr. Soc.* **4**, bvz039 (2020). 32099951 10.1210/jendso/bvz039 PMC7033038

[12] Hubatsch, I., Ragnarsson, E. G. & Artursson, P. Determination of drug permeability and prediction of drug absorption in Caco-2 monolayers. *Nat. Protoc.* **2**, 2111–2119 (2007). 17853866 10.1038/nprot.2007.303

[13] Gupta, V., Doshi, N. & Mitragotri, S. Permeation of insulin, calcitonin and exenatide across Caco-2 monolayers: measurement using a rapid, 3-day system. *PLoS ONE* **8**, e57136 (2013). 23483881 10.1371/journal.pone.0057136 PMC3586668

[14] Lozoya-Agullo, I. et al. Usefulness of Caco-2/HT29-MTX and Caco-2/HT29-MTX/Raji B coculture models to predict intestinal and colonic permeability compared to Caco-2 monoculture. *Mol. Pharm.* **14**, 1264–1270 (2017). 28263609 10.1021/acs.molpharmaceut.6b01165

[15] Stillhart, C. et al. Impact of gastrointestinal physiology on drug absorption in special populations—an UNGAP review. *Eur. J. Pharm. Sci.* **147**, 105280 (2020). 32109493 10.1016/j.ejps.2020.105280

[16] Kwon, O. et al. The development of a functional human small intestinal epithelium model for drug absorption. *Sci. Adv.* **7**, eabh1586 (2021). 10.1126/sciadv.abh1586 PMC11210309 34078609

[17] Ivanov, A. I. Structure and regulation of intestinal epithelial tight junctions: current concepts and unanswered questions. *Adv. Exp. Med. Biol.* **763**, 132–148 (2012). 23397622 10.1007/978-1-4614-4711-5_6

[18] Camilleri, M. Leaky gut: mechanisms, measurement and clinical implications in humans. *Gut* **68**, 1516–1526 (2019). 31076401 10.1136/gutjnl-2019-318427 PMC6790068

[19] Turner, J. R. Intestinal mucosal barrier function in health and disease. *Nat. Rev. Immunol.* **9**, 799–809 (2009). 19855405 10.1038/nri2653

[20] Dumontet, C. & Jordan, M. A. Microtubule-binding agents: a dynamic field of cancer therapeutics. *Nat. Rev. Drug Discov.* **9**, 790–803 (2010). 20885410 10.1038/nrd3253 PMC3194401

[21] Jordan, M. A. & Wilson, L. Microtubules as a target for anticancer drugs. *Nat. Rev. Cancer* **4**, 253–265 (2004). 15057285 10.1038/nrc1317

[22] Jung, K. B. et al. Interleukin-2 induces the in vitro maturation of human pluripotent stem cell-derived intestinal organoids. *Nat. Commun.* **9**, 3039 (2018). 30072687 10.1038/s41467-018-05450-8 PMC6072745

[23] Kwon, O. et al. Chemically-defined and scalable culture system for intestinal stem cells derived from human intestinal organoids. *Nat. Commun.* **15**, 799 (2024). 38280855 10.1038/s41467-024-45103-7 PMC10821882

[24] Krndija, D. et al. Active cell migration is critical for steady-state epithelial turnover in the gut. *Science* **365**, 705–710 (2019). 31416964 10.1126/science.aau3429

[25] Lee, H. et al. Limosilactobacillus reuteri DS0384 promotes intestinal epithelial maturation via the postbiotic effect in human intestinal organoids and infant mice. *Gut Microbes* **14**, 2121580 (2022). 36130031 10.1080/19490976.2022.2121580 PMC9519030

[26] Srinivasan, B. et al. TEER measurement techniques for in vitro barrier model systems. *J. Lab. Autom.* **20**, 107–126 (2015). 25586998 10.1177/2211068214561025 PMC4652793

[27] Yang, H. et al. The role of cellular reactive oxygen species in cancer chemotherapy. *J. Exp. Clin. Cancer Res.* **37**, 266 (2018). 30382874 10.1186/s13046-018-0909-x PMC6211502

[28] Konsoula, R. & Barile, F. A. Correlation of in vitro cytotoxicity with paracellular permeability in Caco-2 cells. *Toxicol. In Vitro* **19**, 675–684 (2005). 15896555 10.1016/j.tiv.2005.03.006

[29] Zoio, P., Lopes-Ventura, S., Marto, J. & Oliva, A. Open-source human skin model with an in vivo-like barrier for drug testing. *ALTEX* **39**, 405–418 (2022). 35319071 10.14573/altex.2111182

[30] Florenes, V. A. et al. A Three-dimensional ex vivo viability assay reveals a strong correlation between response to targeted inhibitors and mutation status in melanoma lymph node metastases. *Transl. Oncol.* **12**, 951–958 (2019). 31096111 10.1016/j.tranon.2019.04.001 PMC6520638

[31] Byron Cryer, M. F. Cyclooxygenase-1 and cyclooxygenase-2 selectivity of widely used nonsteroidal anti-inflammatory drugs. *Am. J. Med.* **104**, 413–421 (1998). 9626023 10.1016/s0002-9343(98)00091-6

[32] Peters, M. F. et al. Human 3D gastrointestinal microtissue barrier function as a predictor of drug-induced diarrhea. *Toxicol. Sci.* **168**, 3–17 (2019). 30364994 10.1093/toxsci/kfy268 PMC6390652

[33] Yu, H. et al. Prospective pharmacological methodology for establishing and evaluating anti-cancer drug resistant cell lines. *BMC Cancer* **21**, 1049 (2021). 34560848 10.1186/s12885-021-08784-7 PMC8464141

[34] Kruidenier, L., Kuiper, I., Lamers, C. B. & Verspaget, H. W. Intestinal oxidative damage in inflammatory bowel disease: semi-quantification, localization, and association with mucosal antioxidants. *J. Pathol.* **201**, 28–36 (2003). 12950014 10.1002/path.1409

[35] Was, H. et al. Mechanisms of chemotherapy-induced neurotoxicity. *Front. Pharmacol.* **13**, 750507 (2022). 35418856 10.3389/fphar.2022.750507 PMC8996259

[36] Zhou, L. et al. Rapamycin prevents cyclophosphamide-induced over-activation of primordial follicle pool through PI3K/Akt/mTOR signaling pathway in vivo. *J. Ovarian Res.* **10**, 56 (2017). 10.1186/s13048-017-0350-3 PMC5559863 28814333

[37] Pike, C. M. et al. High-throughput assay for predicting diarrhea risk using a 2D human intestinal stem cell-derived model. *Toxicol. In Vitro* **106**, 106040 (2025). 40086646 10.1016/j.tiv.2025.106040 PMC12412184

[38] Lechuga, S., Braga-Neto, M. B., Naydenov, N. G., Rieder, F. & Ivanov, A. I. Understanding disruption of the gut barrier during inflammation: should we abandon traditional epithelial cell lines and switch to intestinal organoids? *Front. Immunol.* **14**, 1108289 (2023). 36875103 10.3389/fimmu.2023.1108289 PMC9983034

[39] Chu, S. W., Moujaber, O., Lemay, S. & Stochaj, U. Multiple pathways promote microtubule stabilization in senescent intestinal epithelial cells. *NPJ Aging* **8**, 16 (2022). 10.1038/s41514-022-00097-8 PMC9758230 36526654

[40] Kim, Y. J. et al. Links of cytoskeletal integrity with disease and aging. *Cells* **11**, 2896 (2022). 36139471 10.3390/cells11182896 PMC9496670

[41] Wilms, E. et al. Intestinal barrier function is maintained with aging—a comprehensive study in healthy subjects and irritable bowel syndrome patients. *Sci. Rep.* **10**, 475 (2020). 31949225 10.1038/s41598-019-57106-2 PMC6965102

[42] Sarangi, U., Paithankar, K. R., Kumar, J. U., Subramaniam, V. & Sreedhar, A. S. 17AAG treatment accelerates doxorubicin induced cellular senescence: Hsp90 interferes with enforced senescence of tumor cells. *Drug Target Insights* **6**, 19–39 (2012). 22915839 10.4137/DTI.S9943 PMC3422084

[43] Dincer, Y. et al. Oxidative DNA damage and antioxidant activity in patients with inflammatory bowel disease. *Dig. Dis. Sci.* **52**, 1636–1641 (2007). 17393334 10.1007/s10620-006-9386-8

[44] Balmus, I. M., Ciobica, A., Trifan, A. & Stanciu, C. The implications of oxidative stress and antioxidant therapies in inflammatory bowel disease: clinical aspects and animal models. *Saudi J. Gastroenterol.* **22**, 3–17 (2016). 26831601 10.4103/1319-3767.173753 PMC4763525

[45] Chernyavskij, D. A., Galkin, I. I., Pavlyuchenkova, A. N., Fedorov, A. V. & Chelombitko, M. A. Role of mitochondria in intestinal epithelial barrier dysfunction in inflammatory bowel disease. *Mol. Biol.* **57**, 1024–1037 (2023). 10.31857/S0026898423060058 38062958

[46] Saito, Y. et al. Establishment of patient-derived organoids and drug screening for biliary tract carcinoma. *Cell Rep.* **27**, 1265–1276. e1264 (2019). 31018139 10.1016/j.celrep.2019.03.088

[47] Zhao, Q., Wu, Z. E., Li, B. & Li, F. Recent advances in metabolism and toxicity of tyrosine kinase inhibitors. *Pharmacol. Ther.* **237**, 108256 (2022). 35901905 10.1016/j.pharmthera.2022.108256

[48] Harirforoosh, S., Asghar, W. & Jamali, F. Adverse effects of nonsteroidal antiinflammatory drugs: an update of gastrointestinal, cardiovascular and renal complications. *J. Pharm. Pharm. Sci.* **16**, 821–847 (2013). 24393558 10.18433/j3vw2f

[49] Ryu, B. et al. Next-generation intestinal toxicity model of human embryonic stem cell-derived enterocyte-like cells. *Front. Vet. Sci.* **8**, 587659 (2021). 34604364 10.3389/fvets.2021.587659 PMC8481684

