# Supplementary material for: Drug-induced gastrointestinal toxicity and barrier integrity: cytoskeleton-mediated impairment in a clinically relevant human intestinal epithelium model
Source: Exp Mol Med. 2026 Feb 12;58(2):487–500. doi: 10.1038/s12276-025-01635-6 (PMC12992830; doi:10.1038/s12276-025-01635-6)
Supplement: Supplementary file 1 — Supplementary Information [file 12276_2025_1635_MOESM1_ESM.pdf]

Supplementary Figures

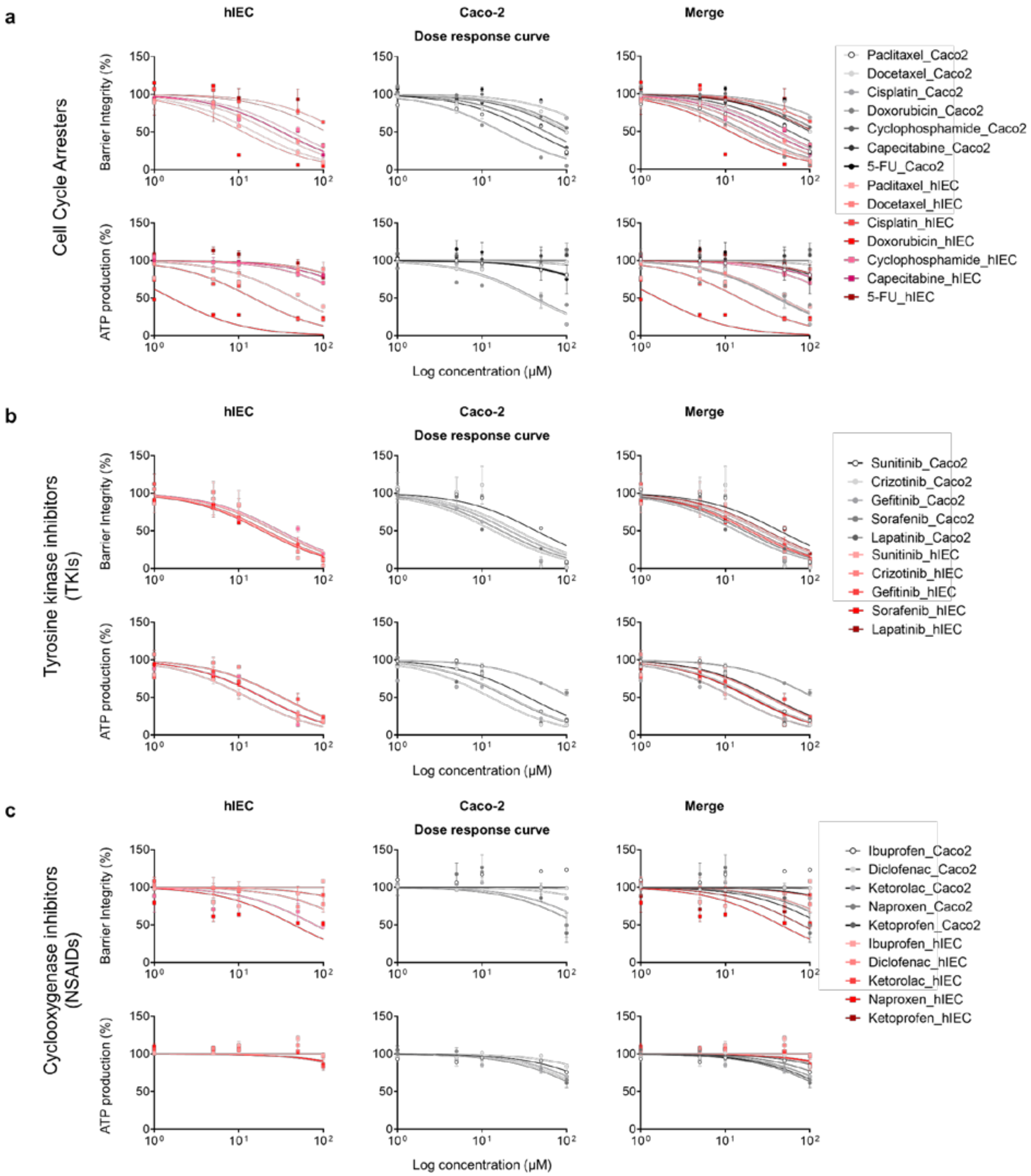

Supplementary Fig. 1. Summary of drug-screening results according to the drug class: (a) cell cycle arresters, (b) TKIs, and (c) NSAIDs analyzed by transepithelial electrical resistance (TEER) and cell-viability data with both human intestinal epithelial cells (hIEC) and Caco-2 cell models.

The y-axis indicates the normalized barrier integrity (%) for the TEER assays and ATP production (%) for the cell viability assays. The X-axis shows the drug concentration (log scale, μм).

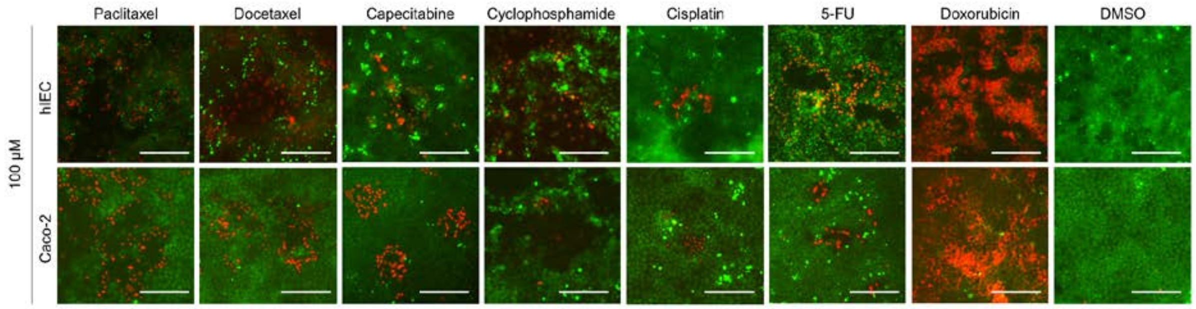

Supplementary Fig. 2. Cell cycle arresters caused severe cell death and senescent metabolism

Representative images from live-dead cell-staining assays with human intestinal epithelial cell (hIEC) and Caco-2 models treated with cell cycle arresters at 100 μM. Cell images were taken at 20X magnification. Scale bar: 200 μm.

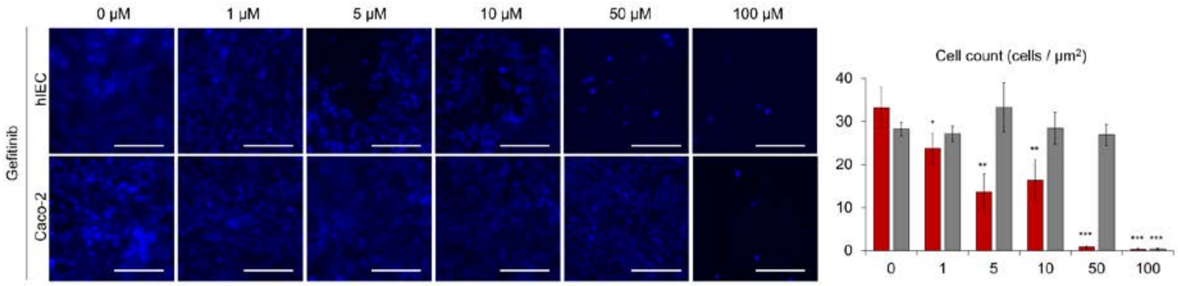

Supplementary Fig. 3. Dose-dependent changes of DAPI-positive cells following gefitinib treatment

Representative DAPI images and corresponding quantitative analyses of DAPI-positive cells in hIECs and Caco-2 cells. Cell images were taken at 20X magnification. Scale bar: 200 μm.

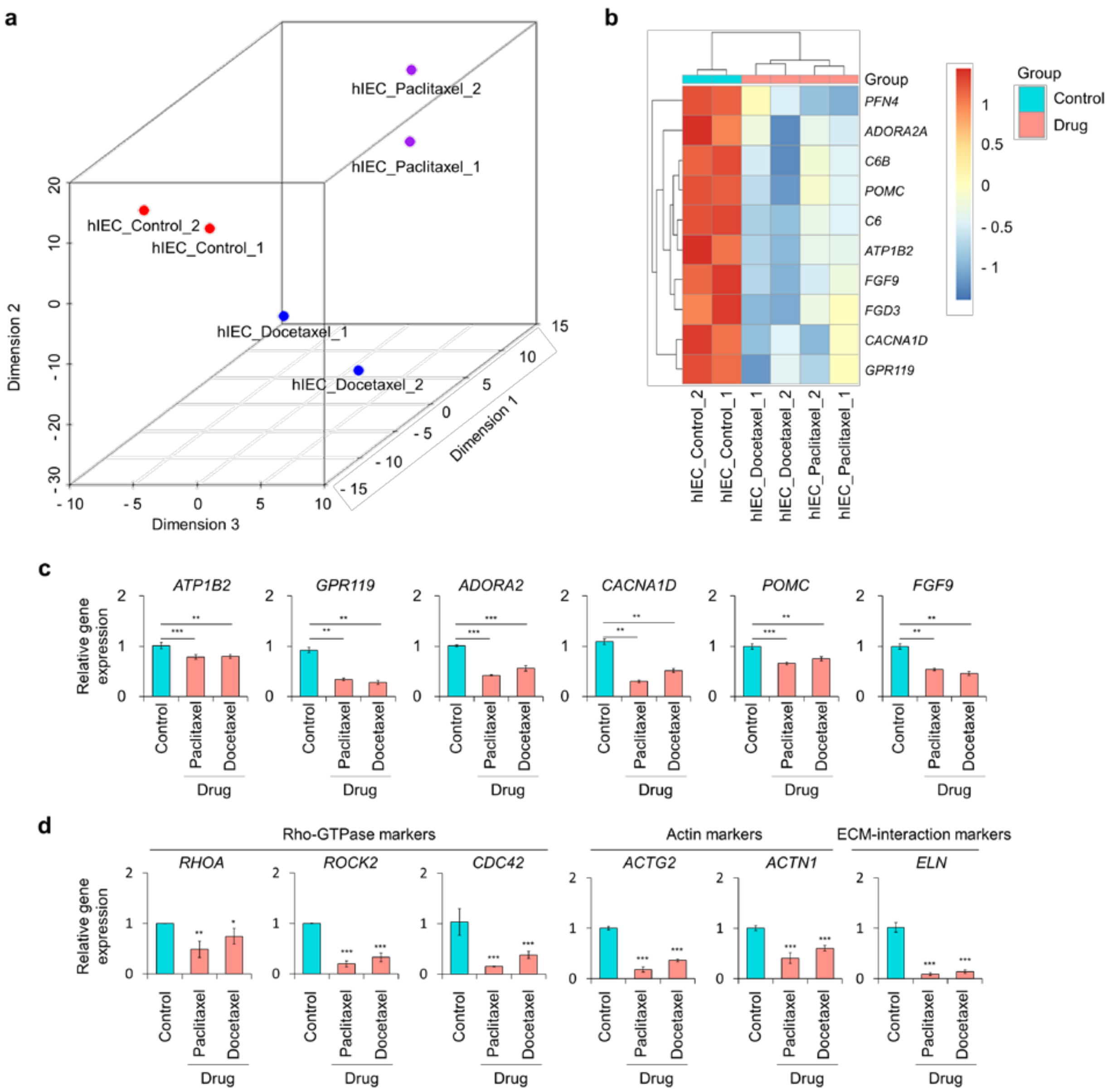

Supplementary Fig. 4. Transcriptomic profiling and qPCR validation of selected functional genes in drug-treated IECs. (a) 3D MDS plot showing clear separation between control and drug-treated hIECs based on transcriptomic profiles of selected functional genes. (b) Heatmap of representative genes associated with intestinal barrier function, signaling, and metabolism, demonstrating consistent downregulation across both paclitaxel- and docetaxel-treated groups relative to controls. (c) qPCR validation of the selected genes confirming significant downregulation in drug-treated hIECs compared to controls. (d) qPCR validation of Rho-GTPase, actin, and junction-related genes (*RHOA*, *ROCK2*, *CDC42*, *ACTG1*, *ACTN1*, and *ELN*)

show decreased expression compared with controls. Statistical significance was determined by conducting a t-test. Data are presented as mean ± (SEM); * $p < 0.05$, ** $p < 0.01$, *** $p < 0.001$

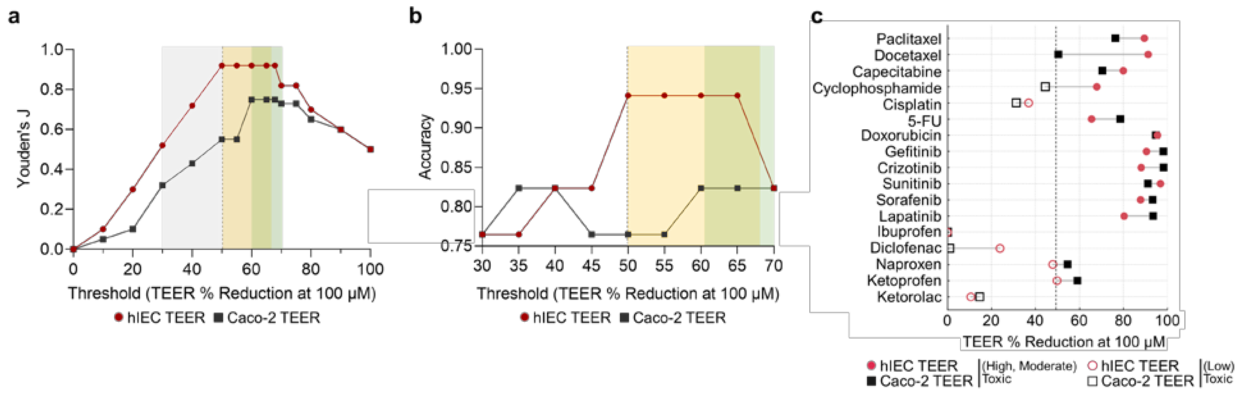

Supplementary Fig. 5. Threshold analysis of the hIEC TEER and Caco-2 TEER models. (a) Youden's J plotted against percent TEER reduction thresholds (0–100 %). The shaded gray area (30–70%) represents the main cutoff analysis window. The yellow box (50–65%) indicates the plateau range for the hIEC TEER model (J ≈ 0.92), while the green box (60–70%) denotes the plateau for the Caco-2 TEER model (J ≈ 0.75). (b) Accuracy plotted across percent TEER reduction thresholds (0–100%). The yellow (hIECs) and green (Caco-2 cells) boxes indicate the same plateau regions defined in panel (a), and the vertical dashed line at 50% marks the adopted percent reduction threshold used for classification. (c) Paired scatter plots comparing percent TEER reduction for individual drugs between hIEC (red) and Caco-2 (black) models; each line connects the paired values for a single drug.

Supplementary Tables

Supplementary Table 1. Sequences of primers used in this study

Gene name	Primer (Forward)	Primer (Reverse)
GAPDH	GAAGGTGAAGGTCGGAGTC	GAAGATGGTGATGGGATTTC
VIL1	TACCTGCTCTACGTTTGGCA	CATGCGTCCCTTGAAGATGG
MUC2	GACCACAACGATTCCTACGC	ACTGCCATCGGACTTGAAGA
MUC13	CGGCTACCAGGAAGATGCTA	TGAGAATGACAATGCCAGCG
CHGA	GACACACTTTCCAAGCCCAG	CGCTGTGTTTCTTCTGCTGA
ECAD	CTCCCTACGACTCTCTGCTG	CTCCTCCATACATGTCCGCT
ZO-1	TCTTCGCAGCTCCAAGAGAA	AGGCCTCAGAAATCCAGCTT
OCLN	GAGACTACAGGTGCCCATCA	CACACACACACACTTAGGCC
CLDN1	TGCTTGGAAGACGATGAGGT	CAGTGAAGAGAGCCTGACCA
CYP3A4	GGCACCACCCACCTATGATA	ATCATCACCACCACCCCTTT
OATP1A2	AGACCAACGCAGGATCCAT	GAGTTTCACCCATTCCACGTACA
LAMA1	CAGAACCAAGCAGACTACGC	TTCCATCTCCCACCACAGTC
COL3A1	TGGCATCAAAGGACATCGAG	TCCACTGGTTCCATCTTTGC
CLDN16	CCTTCCACCTTATGCACACA	GAACCAAAAGCCAGGGAGAA
CLDN19	CCAGATCTCAAGAACGCAGC	TTGTGTCATTCCTCCCCTCC
GIP	ACTTTGTGAACTGGCTGCTG	TCTTCATCGCTGGGGTTCTT
GPR119	GAGTGATCCTTGCTGTCCTG	GGAGGAAGTGACAAATGCCA
SPTBN4	GAGTGGAAGGACGGACTGAA	AGGTCATGCTCAAAGGCTCT
SPP1	GGCTAAACCCTGACCCATCT	CCGTGGGAAAATCAGTGACC
ATP1B2	TTTTCCCTCTTTGCTGGCAC	AGAATCGCTTGAACCTGGGA
ADORA2A	CATTGACCGCTACATTGCCA	GTGGTTCTTGCCCTCCTTTG
CACNA1D	GCATGTGTCTGAAAATGGGC	GGGTCCCTGAAATAGCCATG
POMC	CTGGAGAGCAGCCAGTGTCAG	AGAGGCTGCTCGTCGCCATTTC
FGF9	CCA GGA AAG ACC ACA GCC GAT T	CCA TAC AGC TCC CCC TTC TCA T
RHOA	GTGGCAGATATCGAGGTGGA	ACTATCAGGGCTGTCGATGG
ROCK2	GAGGAGTTCAAGACCAGCCT	TGGGTTCAAGCGATTCTCCT
CDC42	TGCAAACGGTCAGGGATACT	AATGCCAAGTTGTTTCGCCT
ACTG2	TGTGTGAAGAGGAGACCACC	TGTAGAAGGAGTGGTGCCAG
ACTN1	CTTCGACAACAAGCACACCA	TAACCCAAGCTGATGAGGCA
ELN	CAAGACCTGGCTTCGGATTG	TTTCCTTGCCCTGTGGATCT

Supplementary Table 2. Antibodies used in this study

Primary Antibody	Species	Concentration	Source	Catalog number	RRID	Used in
VIL1	Mouse	1:100	Cell signaling	55883S	N/A	Immuno-staining
MUC2	Mouse	1:50	Cell signaling	88686S	N/A	Immuno-staining
CDX2	Rabbit	1:100	Cell signaling	23083SF	N/A	Immuno-staining
CYP3A4	Mouse	1:100	Thermo Fisher	MA5-17064	AB_2538535	Immuno-staining
ZO-1	Rabbit	1:100	Thermo Fisher	61-7300	AB_2533938	Immuno-staining
ECAD	Rabbit	1:100	Cell signaling	3195S	AB_2291471	Immuno-staining
OATP1A2	Rabbit	1:100	Thermo Fisher	PA5-42445	AB_2608733	Immuno-staining
Na^+-K^+ ATPase	Rabbit	1:100	Abclonal	A11683	AB_2861628	Immuno-staining
**Secondary Antibody**	**Species**	**Concentration**	**Source**	**Catalog number**	**RRID**	**Used in**
Anti-Mouse 488	Goat	1:200	Thermo Fisher	A21131	AB_2535771	Immuno-staining
Anti-Mouse 594	Donkey	1:200	ThermoF isher	A21203	AB_2535789	Immuno-staining
Anti-Rabbit 488	Chicken	1:200	Thermo Fisher	A21441	AB_2535859	Immuno-staining
Anti-Rabbit 594	Chicken	1:200	Thermo Fisher	A21442	AB_2535860	Immuno-staining

Supplementary Table 3. Total GI-toxicity scoring criteria

GI-symptom severity (grades 1–4) and symptom incidence (very common = 3, common = 2, uncommon = 1) scores were used to determine the drug GI-toxicity categories. The data presented are associated with Table 2.

**Grade score**	**GI symptoms severity grade**
4	Life-threatening (hemorrhagic colitis, perforation); urgent medical intervention
3	Severe diarrhea, severe mucositis affecting diet, hospitalization required; clear intestinal barrier disruption
2	Moderate diarrhea, mild/moderate mucositis; minimal medical intervention (oral hydration or antidiarrheals)
1	Mild diarrhea, slight mucosal irritation; no clinical intervention.
**Incidence score**	**Incidence classification**
3	Very common (≥10%)
2	Common (1–10%)
1	Uncommon (<1%)
**Total GI toxicity score**	**Clinical toxicity level**
≥ 9	High toxicity (Toxic)
4–8	Moderate toxicity (Toxic)
≤ 3	Low toxicity

Supplementary Table 4. Raw and normalized TEER and ATP cell-viability values from intestinal-toxicity assays (separate file)

Absolute and normalized values (mean ± SEM) from the TEER and ATP-based cell-viability assays using hIEC and Caco-2 cells at increasing drug concentrations (0, 1, 5, 10, 50, and 100 μᴍ). The data shown represent four independent replicate experiments and are associated with Figure 2 and Table 3.

Supplementary Table 5. C_{max} and MOS (IC_{15}/C_{max}) values for drugs evaluated in intestinal toxicity assays

Median [IQR] and min–max values of C_{max} (µM) were obtained from peer-reviewed literature and FDA regulatory sources for each drug. MOS values were calculated from hIEC-based TEER IC_{15} data (IC_{15}:C_{max}) to reflect exposure variability and pharmacokinetic context. Abbreviated sources (journal, year, PMID, and/or FDA application, label, and DrugBank ID) are listed within the table; full references are available upon request. When only a single C_{max} reports was available, dispersion metrics (IQR and min–max) and corresponding MOS ranges were denoted as NA.

Drug (Route)	MW (g/mol)	IC_{15} (µM)*	Clinical C_{max} median [IQR] (µᴍ)	C_{max} min–max (µᴍ)	MOS (IC_{15}/C_{max} median (min–max))	References
Paclitaxel (IV infusion)	853.91	2.71	5.1 [4.6–5.8]	3.8–6.2	0.53 (0.44–0.71)	Clin Pharmacokinet 2021; PMID 28612269; DB01229
Docetaxel (IV infusion)	807.88	4.36	5.1 [4.8–5.6]	4.0–6.0	0.85 (0.73–1.09)	Clin Cancer Res 2004; PMID 15041715; DB01248
Capecitabine (PO BID)	359.35	5.74	13.7 [12.8–14.5]	10.0–16.0	0.42 (0.36–0.57)	Clin Pharmacokinet 2001; PMID 11286326; FDA XELODA label (NDA 020896); DB01101
Cyclophosphamide (PO QD)	261.09	7.40	122.0 [110–135]	90.0–150.0	0.06 (0.05–0.08)	Eur J Cancer 2016; PMID 26773420; ClinicalTrials.gov Identifier NCT00334646; DB00531
Cisplatin (IV)	300.05	30.49	14.8 [13.0–16.3]	10.0–18.0	2.06 (1.69–3.05)	J Clin Diagn Res 2016; PMID 27630935; Eur J Cancer 2022; PMID 34810046; DB00515
5-Fluorouracil (IV bolus)	130.08	19.31	34.1 [31.2–36.9]	25.0–45.0	0.57 (0.43–0.78)	Pharmacol Res 2004; PMID 15177306; Clin Cancer Res 2000; PMID 10955781; DB00544
Doxorubicin (IV)	543.52	1.99	7.6 (high-dose end-inf.; no IQR/min–max)	NA	0.26	Eur J Pharm Sci 2010; PMID 20688160; DB00997

Gefitinib (PO QD)	446.90	3.80	0.5 [0.4–0.6]	0.3–0.7	7.76 (5.43–12.70)	Lung Cancer 2016; PMID 26898617; Transl Clin Pharmacol 2021; PMID 34621709; DB00317
Crizotinib (PO BID)	450.34	4.81	1.0	0.7–1.6	4.81 (3.01–6.87)	FDA XALKORI label (250 mg BID, steady-state; NDA 202570); DB08865
Sunitinib (PO QD, total)	398.47	3.68	0.3 [0.3–0.4]	0.3–0.4	11.20 (9.30–13.50)	FDA SUTENT label (parent + SU12662; NDA 021938); DB01268
Sorafenib (PO BID)	464.83	3.36	6.5 [5.9–7.1]	5.0–8.0	0.52 (0.45–0.66)	Oncologist 2007; PMID 17470685; Pharmacogenet Genomics 2018; PMID 28362716; FDA NEXAVAR label (NDA 021923); DB00398
Lapatinib (PO QD)	581.06	5.44	4.2 [3.8–4.7]	3.0–5.0	1.30 (1.05–1.65)	FDA TYKERB label (NDA 022059); DB01259
Ibuprofen (PO)	206.28	>100	145.0	–	>0.69	BMC Clin Pharmacol 2009; PMID 19961574; DB01050
Diclofenac (PO)	296.15	43.52	5.0	4.0–6.0	8.70	Br J Cancer 2012; PMID 22531634; DB00586
Naproxen (PO)	230.26	7.90	313.0	280.0–340.0	0.03 (0.02–0.03)	Clin Ther 1994; PMID 7697688; DB00788
Ketoprofen (PO)	254.28	14.11	10.8	8.0–13.0	1.31 (1.09–1.76)	FDA Application No. 022470 (Orudis® tablets); Br J Clin Pharmacol 1995; PMID 7768385; DB01009
Ketorolac (PO 10 mg)	255.27	134.29	11.0	9.9–11.7	12.20 (11.50–13.60)	FDA TORADOL label (10 mg PO tablet; NDA 019645); DB00465

* IC_{15} values were obtained from Table 3 (hIEC-based TEER assay) and used to compute MOS.

[1] MOS threshold. MOS ($IC_{15}/C_{max} < 1$) was used as a conservative clinical risk threshold; MOS ≥ 1 denotes non-toxic exposure margin.

[2] Bracket notation for C_{max} in S5. [a–b] = IQR (interquartile range); (a–b) = min–max range; no brackets = single reported value (dispersion data unavailable).

Examples: 4.2 [3.8–4.7] µM → median 4.2, IQR 3.8–4.7; 5.0 (4.0–6.0) µM → min 4, max 6.

[3] Infusion drugs (e.g., doxorubicin). C_{max} varies with dose ($mg \cdot m^{-2}$) and infusion duration (bolus vs 30–90 min). The 7.6 µM value reflects a high-dose, end-of-infusion condition; lower medians (~1.5 µM at 60–75 $mg \cdot m^{-2}$) are reported and would shift MOS (IC_{15}/C_{max}) for hIEC-TEER from TP → FN, while ≥50% TEER reduction classification remains unchanged.

[4] Crizotinib. Primary C_{max} = 1.0 µM (range 0.7–1.6) based on label steady-state 250 mg BID.
[5] Sunitinib. C_{max} reflects total exposure (parent + active metabolite SU12662) per label.
[6] Ketoprofen. C_{max} varies with food intake (fed vs. fasted ≈ 9–16 µM); median 10.8 µM shown.
[7] Ketorolac. C_{max} from oral 10 mg tablet in healthy adults (2.99 µg·mL^{-1} ≈ 11.7 µM; elderly ≈ 9.9 µM); *fasted single-dose PK* ≈ 3.4 µM (2.7–4.3), used only for sensitivity; MOS classification unaffected.

Abbreviations. MW, molecular weight; IC_{15}, 15% inhibitory concentration (TEER); C_{max}, maximum observed plasma concentration; MOS, margin of safety; PO, per os (oral); IV, intravenous; QD, once daily; BID, twice daily; IQR, interquartile range; NA, not available / not applicable.

Supplementary Table 6. Incidence and within-class variability of severe gastrointestinal (GI) toxicity (grade ≥3) among the analyzed drugs

GI toxicity information (e.g., diarrhea, mucositis, colitis, ileitis, perforation) was extracted from FDA labels and guidelines as well as curated sources (DrugBank, Drugs.com, Mayo Clinic) for short-course, fixed-dose regimens in patients without pre-existing GI disease, and was compared with model outcomes. To assess within-class clinical heterogeneity, the columns show clinical toxicity levels (Table 2) and report concordance with the hIEC-based TEER classifier (≥50% reduction threshold).

Drug	Grade 3 ≥ GI incidence	Clinical toxicity level	Within-class comparison / TEER concordance
**Cell cycle arresters**			
Paclitaxel	~ 5 %	Moderate	Correctly classified toxic
Docetaxel	~ 5–10 %	High	Correctly toxic
Capecitabine	~ 11 %	High	High within class; correctly toxic
Cyclophosphamide	< 1 %	Moderate	Borderline; correctly toxic
Cisplatin	≈ 0 %	Low	Lowest within class; correctly non-toxic
5-Fluorouracil	5–15 %	High	Highest within class; correctly toxic
Doxorubicin	< 5 %	Moderate	Intermediate; correctly toxic
**TKIs**			
Gefitinib	≈ 3 %	Moderate	Correctly toxic
Crizotinib	< 5 %	Moderate	Correctly toxic
Sunitinib	3–5 %	Moderate	Correctly toxic
Sorafenib	1–5 %	Moderate	Correctly toxic
Lapatinib	5–10 %	High	Highest within TKIs; correctly toxic
**NSAIDs**			
Ibuprofen	< 1 %	Low	Correctly non-toxic
Diclofenac	< 1 %	Low	Correctly non-toxic

Naproxen	< 1 %	Low	Correctly non-toxic
Ketoprofen	< 1 %	Low	Correctly non-toxic
Ketorolac	< 1 % (uncommon)	Moderate	Slightly higher NSAID; false-negative (FN) case

Supplementary Table 7. Drug classification into TPs, TNs, FPs, and FNs based on percent reduction and MOS

Drugs showing true positive (TP), true negative (TN), false positive (FP), and false negative (FN) results according to the percent reduction (≥50%) and margin of safety (MOS, IC_{15}:C_{max} < 1) thresholds were used in the intestinal toxicity assays. The data correspond to Fig. 5, with additional columns displaying the AUC (95% CI, DeLong) and Mann–Whitney p-values for each assay, thereby enhancing transparency in diagnostic accuracy reporting.

**% Reduction Assay**	**TP**	**TN**	**FP**	**FN**	**AUC (95% CI, DeLong)**	**Mann–Whitney *p***
hIEC TEER	Paclitaxel, Docetaxel, Capecitabine, Cyclophosphamide, 5-FU, Doxorubicin, Gefitinib, Crizotinib, Sunitinib, Sorafenib, Lapatinib	Cisplatin, Ibuprofen, Diclofenac, Naproxen, Ketoprofen	None	Ketorolac	0.96 (0.82–1.00)	<0.001
Caco-2 TEER	Paclitaxel, Docetaxel, Capecitabine, 5-FU, Doxorubicin, Gefitinib, Crizotinib, Sunitinib, Sorafenib, Lapatinib	Cisplatin, Ibuprofen, Diclofenac	Naproxen‡, Ketoprofen‡	Cyclophosphamide†, Ketorolac	0.72 (0.48–0.89)	0.036
hIEC ATP	Paclitaxel, Doxorubicin, Gefitinib, Crizotinib, Sunitinib, Sorafenib, Lapatinib	Ibuprofen, Diclofenac, Naproxen, Ketoprofen	Cisplatin	Docetaxel, Capecitabine, Cyclophosphamide, 5-FU, Ketorolac	0.69 (0.44–0.88)	0.049
Caco-2 ATP	Doxorubicin, Gefitinib, Crizotinib, Sunitinib, Lapatinib	Ibuprofen, Diclofenac, Naproxen, Ketoprofen	Cisplatin	Paclitaxel, Docetaxel, Capecitabine,	0.61 (0.37–0.81)	0.092

				Cyclophosphamide, 5-FU, Sorafenib, Ketorolac		

**MOS (IC_{15}/Cmax) Assay**	**TP**	**TN**	**FP**	**FN**	**AUC (95% CI, DeLong)**	**Mann–Whitney *p***
hIEC TEER	Paclitaxel, Docetaxel, Capecitabine, Cyclophosphamide, 5-FU, Doxorubicin, Sorafenib	Cisplatin, Ibuprofen, Diclofenac, Ketoprofen	Naproxen	Gefitinib, Crizotinib, Sunitinib, Lapatinib, Ketorolac	0.69 (0.43–0.90)	0.042
Caco-2 TEER	Capecitabine, Cyclophosphamide, 5-FU, Doxorubicin, Sorafenib, Lapatinib	Cisplatin, Ibuprofen, Diclofenac, Ketoprofen	Naproxen	Paclitaxel, Docetaxel, Gefitinib, Crizotinib, Sunitinib, Ketorolac	0.65 (0.40–0.85)	0.059
hIEC ATP	Cyclophosphamide, Doxorubicin, Sorafenib, Lapatinib	Ibuprofen, Diclofenac, Ketoprofen	Cisplatin, Naproxen	Paclitaxel, Docetaxel, Capecitabine, 5-FU, Gefitinib, Crizotinib, Sunitinib, Ketorolac	0.54 (0.32–0.74)	0.214
Caco-2 ATP	Doxorubicin, Lapatinib	Diclofenac, Ketoprofen	Cisplatin, Ibuprofen, Naproxen	Paclitaxel, Docetaxel, Capecitabine, Cyclophosphamide, 5-FU, Gefitinib, Crizotinib, Sunitinib, Sorafenib, Ketorolac	0.40 (0.22–0.58)	0.308

† Compounds classified as TPs in the hIEC TEER assay but as FNs in the Caco-2 TEER assay (≥50% TEER reduction at 100 μM). These cases demonstrate the sensitivity of the hIEC model to detect clinically relevant epithelial injury.

‡ Compounds classified as FPs in the Caco-2 TEER assay (≥50% TEER reduction at 100 μM) despite low GI toxicity (see Tables 2 and S6).

§ Compounds classified as FNs in both TEER assays (ketorolac), reflecting a mechanism not associated with early decrease in TEER (prostaglandin

inhibition/mucosal irritation).
Clinical toxicity levels were assigned as in Tables 2 and S6.

Supplementary Table 8. Diagnostic-accuracy metrics based on the MOS (IC_{50}:Cmax ratio <1) for intestinal toxicity assays

The sensitivity, specificity, accuracy, PPV, NPV, ROC AUC, and drug classification (TPs, TNs, FPs, and FNs) were calculated based on the MOS threshold (IC_{50}:Cmax ratio <1), highlighting the limitations of IC_{50} determinations as an early diagnostic indicator. The data shown are associated with Figure 2 and Table 3.

MOS (IC_{50}/Cmax) Assay	TP	TN	FP	FN	Sensitivity	Specificity	Accuracy	PPV	NPV	AUC
hIEC TEER	1	4	1	11	0.08	0.80	0.29	0.50	0.27	0.44
Caco-2 TEER	0	4	1	12	0.00	0.80	0.24	0.00	0.25	0.40
hIEC ATP	1	5	0	11	0.08	1.00	0.35	1.00	0.31	0.54
Caco-2 ATP	0	4	1	12	0.00	0.80	0.24	0.00	0.25	0.40

MOS (IC_{50}/Cmax) Assay	TP	TN	FP	FN
hIEC TEER	Cyclophosphamide	Cisplatin, Ibuprofen, Diclofenac, Ketoprofen	Naproxen	Paclitaxel, Docetaxel, Capecitabine, 5-FU, Doxorubicin, Gefitinib, Crizotinib, Sunitinib, Sorafenib, Lapatinib, Ketorolac
Caco-2 TEER	None	Cisplatin, Ibuprofen, Diclofenac, Ketoprofen	Naproxen	Paclitaxel, Docetaxel, Capecitabine, Cyclophosphamide, 5-FU, Doxorubicin, Gefitinib, Crizotinib, Sunitinib, Sorafenib, Lapatinib, Ketorolac

hIEC ATP	Doxorubicin	Cisplatin, Ibuprofen, Diclofenac, Naproxen, Ketoprofen	None	Paclitaxel, Docetaxel, Capecitabine, Cyclophosphamide, 5-FU, Gefitinib, Crizotinib, Sunitinib, Sorafenib, Lapatinib, Ketorolac
Caco-2 ATP	None	Cisplatin, Ibuprofen, Diclofenac, Ketoprofen	Naproxen	Paclitaxel, Docetaxel, Capecitabine, Cyclophosphamide, 5-FU, Doxorubicin, Gefitinib, Crizotinib, Sunitinib, Sorafenib, Lapatinib, Ketorolac
